# Investigating Optimal Chemotherapy Options for Osteosarcoma Patients through a Mathematical Model

**DOI:** 10.3390/cells10082009

**Published:** 2021-08-06

**Authors:** Trang Le, Sumeyye Su, Leili Shahriyari

**Affiliations:** Department of Mathematics and Statistics, University of Massachusetts Amherst, Amherst, MA 01003, USA; tramle@umass.edu (T.L.); sumeyyesu@umass.edu (S.S.)

**Keywords:** osteosarcoma, data driven mathematical model, immune infiltrations, chemotherapy, precision medicine, optimal dosage, doxorubicin, cisplatin, methotrexate

## Abstract

**Simple Summary:**

Osteosarcoma is a rare type of cancer with poor prognoses. However, to the best of our knowledge, there are no mathematical models that study the impact of chemotherapy treatments on the osteosarcoma microenvironment. In this study, we developed a data driven mathematical model to analyze the dynamics of the important players in three groups of osteosarcoma tumors with distinct immune patterns in the presence of the most common chemotherapy drugs. The results indicate that the treatments’ start times and optimal dosages depend on the unique growth rate of the tumor, which implies the necessity of personalized medicine. Furthermore, the developed model can be extended by others to build models that can recommend individual-specific optimal dosages.

**Abstract:**

Since all tumors are unique, they may respond differently to the same treatments. Therefore, it is necessary to study their characteristics individually to find their best treatment options. We built a mathematical model for the interactions between the most common chemotherapy drugs and the osteosarcoma microenvironments of three clusters of tumors with unique immune profiles. We then investigated the effects of chemotherapy with different treatment regimens and various treatment start times on the behaviors of immune and cancer cells in each cluster. Saliently, we suggest the optimal drug dosages for the tumors in each cluster. The results show that abundances of dendritic cells and HMGB1 increase when drugs are given and decrease when drugs are absent. Populations of helper T cells, cytotoxic cells, and IFN-γ grow, and populations of cancer cells and other immune cells shrink during treatment. According to the model, the MAP regimen does a good job at killing cancer, and is more effective than doxorubicin and cisplatin combined or methotrexate alone. The results also indicate that it is important to consider the tumor’s unique growth rate when deciding the treatment details, as fast growing tumors need early treatment start times and high dosages.

## 1. Introduction

Osteosarcoma is a rare kind of cancer that occurs in the bone, and around 1000 new cases of osteosarcoma are identified each year in the United States [1]. It can affect people of any age, but it mostly occurs in children who are 10 to 14 and in adults who are 65 and older [2,3]. There are some factors such as gender, age, heritable syndromes, and certain other conditions that affect the risk of osteosarcoma [4]. The types of standard treatment for osteosarcoma include surgery, chemotherapy, radiotherapy, and targeted therapy [5].

Although neoadjuvant chemotherapy has shown improved results in treating osteosarcoma, patients with metastases have continued to have low survival rates [6,7,8]. Immunotherapy and targeted therapy are known alternative treatments of osteosarcoma; however, they are still inefficient for many patients [9]. Meanwhile, resistance to radiotherapy has also been observed in osteosarcoma tumors [10,11]. To overcome therapeutic resistance, improve survival rates, and achieve precision medicine, we need to investigate osteosarcoma tumor progression and identify the primary factors in the growth of tumors for each patient [12].

The immune system is one of the major players in the responses to various cancer therapies [13,14,15]. Immune cells in the tumor microenvironment interact with tumor cells directly or indirectly via chemokine and cytokine signaling, and they play critical roles in improving or inhibiting treatment effectiveness and tumor behaviors [16]. The necrotic cell death of tumor cells caused by radiotherapy or chemotherapy triggers the production of high mobility group box 1 (HMGB1), which is a damage-associated molecular pattern (DAMP) molecule, and thus can induce immune responses [17,18,19,20]. HMGB1 can promote dendritic cell maturation from naive dendritic cells [21,22,23,24]. Dendritic cells in turn can activate helper and cytotoxic T cells [25,26,27], leading to the elimination of cancer cells by cytotoxic T cells and IFN-γ [25,27,28], a cytokine secreted by helper and cytotoxic T cells [28,29,30].

Many studies have also reported connections between clinical outcomes and immune cell types in osteosarcoma. Cytotoxic T cells, known as the primary receptors of the immune response targeting ostersarcoma [27], have an important role in the immunological responses of osteosarcoma patients [31]. Additionally, a large number of M1 macrophages in an osteosaroma tumor has been found to be associated with good prognosis in many studies [32,33], and it has been reported that low-risk patients have high numbers of CD8 T cells and NK cells [34]. Moreover, certain chemotherapy drugs, such as cisplatin, can increase the cancer killing capacity of cytotoxic T cells [35,36,37,38], so the effectiveness of the drug also depends on the number of cytotoxic T cells in the tumor microenvironment. Furthermore, most cancer therapies also kill immune cells. Some immune cells have anti-tumor effects and others have pro-tumor effects, so the death of immune cells can have an indirect impact on the growth of tumor. Hence, the complicated relationship between immune cells and tumor cells should be taken into account while studying the impact of a treatment on tumor growth, especially when the treatment affects the immune system.

Most chemotherapy treatments for osteosarcoma include one of or a combination of the following drugs: high-dose methotrexate (MTX), doxorubicin (DOX), and cisplatin (CDDP). The most popular treatment regimen for adolescents is the MAP regimen, consisting of all those three drugs [39,40], and a widely used treatment for older adults is a two-drug regimen of doxorubicin and cisplatin [40]. This study investigated the responses to these regimens by developing a data driven mathematical model which takes into consideration the interactions between tumor-infiltrating immune cells and chemotherapy agents by categorizing patients into groups based on tumor-infiltrating immunological variants.

Mathematical models are commonly used to study the growth of a tumor, to identify the optimal combination of treatments, to improve responses to therapies, and to combat drug resistance in various types of cancer [41,42,43,44,45,46,47,48,49,50,51]. While many of such models exist, only a few focus on the complex interactions between tumor cells and several types of immune cells [52,53,54], and none of them model osteosarcoma. Although there have been some studies that included bone modeling, osteoblast cells, or bone metastases [55,56,57], to the best of our knowledge, our previous work [58] was the first to model the growth of primary osteosarcoma tumors. However, that study did not include the effects of chemotherapy, as it modeled osteosarcoma’s progression in the absence of treatments.

In the above-mentioned study [58], we developed a data driven mathematical model for the interaction network between key immune cells and cancer cells to investigate the growth behavior of three distinct groups of osteosarcoma tumors, grouped based on their immune compositions [33]. Group-specific parameters have been calculated to discover differences in tumor growth among the groups [58]. In this study, we extend our previous model by including the interactions between methotrexate, doxorubicin, cisplatin, and important cell types in the tumor microenvironment in order to examine the effects of these drugs on osteosarcoma tumors in each group.

## 2. Materials and Methods

### 2.1. Mathematical Model

Our previous work [58] developed a comprehensive model of various interactions between the immune system and the osteosarcoma tumor microenvironment. This model includes cancer cells, necrotic cells, macrophages, dendritic cells, cytotoxic cells (cytotoxic T cells and natural killer cells), helper T cells, regulatory T cells, along with IFN-γ, HMGB1, and cytokine groups $\mu_{1}$ and $\mu_{2}$—where $\mu_{1}$ consists of TGF-β, IL-4, IL-10, and IL-13, and $\mu_{2}$ consists of IL-6 and IL-17. In this work, we build upon the model in [58] to inspect the effects of chemotherapy on osteosarcoma, by adding the interactions of chemotherapy drugs with immune and cancer cells.

The interactions among key components of the osteosarcoma tumor microenvironment modeled in [58] are summarized below. IFN-γ, which is secreted by helper T cells and cytotoxic cells [28,29,30], can promote macrophages and inhibit cancer cell proliferation [25,27,28,59,60,61,62]. Cytokine group $\mu_{1}$ is released by helper T cells, macrophages, and cancer cells [30,60,61,62,63,64,65]. $\mu_{1}$ can activate regulatory T cells and macrophages, promote tumor proliferation [29,59,60,62,64,66,67,68,69], and inhibit cytotoxic cells and helper T cells [62,66,70,71]. Cytokine group $\mu_{2}$ is also produced by helper T cells, macrophages, and cancer cells [30,62,63,64,72,73], and can promote tumor proliferation [29,61,67,68,72,73,74]. HMGB1, which is passively secreted by necrotic cells [22,26,75,76] and actively secreted by dendritic cells and macrophages [21,22,23,24,75,77], can activate dendritic cells [21,22,23,24].

Cancer cells activate dendritic cells by releasing tumor antigens [25], but also promote apoptosis in dendritic cells through many tumor-derived factors [78]. Dendritic cells in turn can activate cytotoxic cells and helper T cells by presenting tumor antigens to them [25,26,27], and regulatory T cells inhibit these two cells [25,29,79,80]. Macrophages also activate cytotoxic cells and helper T cells through IL-12 and IL-23 production [25,29,30,64,81,82,83], and helper T cells can directly activate cytotoxic cells as well [25,29]. When cancer cells die and go through necrotic cell death, they become necrotic cells; thus, necrotic cells are promoted by cancer cells.

The model in [58] also included naive T cells, naive macrophages, and naive dendritic cells since mature immune cells differentiate from naive immune cells. The mass action law was used to describe the activation of a cell from its naive form. In particular, for biochemical process A+B→C, the rate of change of *C* is $\frac{dC}{dt}=\lambda AB$, where λ is the production rate of *C* from *A* and *B*. The growth of tumor was modeled using the logistic growth, that is, the rate of change of cancer cell population is proportional to $\left[C\right]\left(1-\frac{\left[C\right]}{C_{0}}\right)$, with $C_{0}$ being the carrying capacity. Cytokines in [58] were modeled to be proportional to the populations of cells that produce them.

The full system of ODEs from the model in [58] is:

(1) $$\begin{array}{cc} \frac{d\left[M_{N}\right]}{dt}=\; & A_{M_{N}}-\left(\lambda_{MI_{\gamma }}\left[I_{\gamma }\right]+\lambda_{M\mu_{1}}\left[\mu_{1}\right]\right)\left[M_{N}\right]-\delta_{M_{N}}\left[M_{N}\right], \end{array}$$

(2) $$\begin{array}{cc} \frac{d\left[M\right]}{dt}=\; & \left(\lambda_{MI_{\gamma }}\left[I_{\gamma }\right]+\lambda_{M\mu_{1}}\left[\mu_{1}\right]\right)\left[M_{N}\right]-\delta_{M}\left[M\right], \end{array}$$

(3) $$\begin{array}{cc} \frac{d\left[T_{N}\right]}{dt}=\; & A_{T_{N}}-\left(\lambda_{T_{h}M}\left[M\right]+\lambda_{T_{h}D}\left[D\right]\right)\left[T_{N}\right]\\ \; & -\lambda_{T_{r}\mu_{1}}\left[\mu_{1}\right]\left[T_{N}\right]\\ \; & -\left(\lambda_{T_{c}T_{h}}\left[T_{h}\right]+\lambda_{T_{c}M}\left[M\right]+\lambda_{T_{c}D}\left[D\right]\right)\left[T_{N}\right]-\delta_{T_{N}}\left[T_{N}\right], \end{array}$$

(4) $$\begin{array}{cc} \frac{d\left[T_{h}\right]}{dt}=\; & \left(\lambda_{T_{h}M}\left[M\right]+\lambda_{T_{h}D}\left[D\right]\right)\left[T_{N}\right]-\left(\delta_{T_{h}T_{r}}\left[T_{r}\right]+\delta_{T_{h}\mu_{1}}\left[\mu_{1}\right]+\delta_{T_{h}}\right)\left[T_{h}\right], \end{array}$$

(5) $$\begin{array}{cc} \frac{d\left[T_{r}\right]}{dt}=\; & \left(\lambda_{T_{r}\mu_{1}}\left[\mu_{1}\right]\right)\left[T_{N}\right]-\delta_{T_{r}}\left[T_{r}\right], \end{array}$$

(6) $$\begin{array}{cc} \frac{d\left[T_{c}\right]}{dt}=\; & \left(\lambda_{T_{c}T_{h}}\left[T_{h}\right]+\lambda_{T_{c}M}\left[M\right]+\lambda_{T_{c}D}\left[D\right]\right)\left[T_{N}\right]\\ \; & -\left(\delta_{T_{c}T_{r}}\left[T_{r}\right]+\delta_{T_{c}\mu_{1}}\left[\mu_{1}\right]+\delta_{T_{c}}\right)\left[T_{c}\right], \end{array}$$

(7) $$\begin{array}{cc} \frac{d\left[D_{N}\right]}{dt}=\; & A_{D_{N}}-\left(\lambda_{DC}\left[C\right]+\lambda_{DH}\left[H\right]\right)\left[D_{N}\right]-\delta_{D_{N}}\left[D_{N}\right], \end{array}$$

(8) $$\begin{array}{cc} \frac{d\left[D\right]}{dt}=\; & \left(\lambda_{DC}\left[C\right]+\lambda_{DH}\left[H\right]\right)\left[D_{N}\right]-\left(\delta_{DC}\left[C\right]+\delta_{D}\right)\left[D\right], \end{array}$$

(9) $$\begin{array}{cc} \frac{d\left[C\right]}{dt}=\; & \left(\lambda_{C}+\lambda_{C\mu_{1}}\left[\mu_{1}\right]+\lambda_{C\mu_{2}}\left[\mu_{2}\right]\right)\left[C\right]\left(1-\frac{\left[C\right]}{C_{0}}\right)\\ \; & -\left(\delta_{CT_{c}}\left[T_{c}\right]+\delta_{CI_{\gamma }}\left[I_{\gamma }\right]+\delta_{C}\right)\left[C\right], \end{array}$$

(10) $$\begin{array}{cc} \frac{d\left[N\right]}{dt}=\; & \alpha_{NC}\left(\delta_{CT_{c}}\left[T_{c}\right]+\delta_{CI_{\gamma }}\left[I_{\gamma }\right]+\delta_{C}\right)\left[C\right]-\delta_{N}\left[N\right], \end{array}$$

(11) $$\begin{array}{cc} \frac{d\left[I_{\gamma }\right]}{dt}=\; & \lambda_{I_{\gamma }T_{h}}\left[T_{h}\right]+\lambda_{I_{\gamma }T_{c}}\left[T_{c}\right]-\delta_{I_{\gamma }}\left[I_{\gamma }\right], \end{array}$$

(12) $$\begin{array}{cc} \frac{d\left[\mu_{1}\right]}{dt}=\; & \lambda_{\mu_{1}T_{h}}\left[T_{h}\right]+\lambda_{\mu_{1}M}\left[M\right]+\lambda_{\mu_{1}C}\left[C\right]-\delta_{\mu_{1}}\left[\mu_{1}\right], \end{array}$$

(13) $$\begin{array}{cc} \frac{d\left[\mu_{2}\right]}{dt}=\; & \lambda_{\mu_{2}T_{h}}\left[T_{h}\right]+\lambda_{\mu_{2}M}\left[M\right]+\lambda_{\mu_{2}C}\left[C\right]-\delta_{\mu_{2}}\left[\mu_{2}\right], \end{array}$$

(14) $$\begin{array}{cc} \frac{d\left[H\right]}{dt}=\; & \lambda_{HM}\left[M\right]+\lambda_{HD}\left[D\right]+\lambda_{HN}\left[N\right]-\delta_{H}\left[H\right]. \end{array}$$

Here, λ parameters denote proliferation, activation, or production rates. δ parameters denote inhibition, decay, or death rates. $A_{M_{N}}$, $A_{T_{N}}$, and $A_{D_{N}}$ correspond to the production/proliferation rates of naive macrophages, naive T cells, and naive dendritic cells, respectively; and $\alpha_{NC}$ corresponds to the fraction of dying cancer cells that become necrotic cells. The descriptions of all variables in this system are given in Table 1.

**Figure 1:**
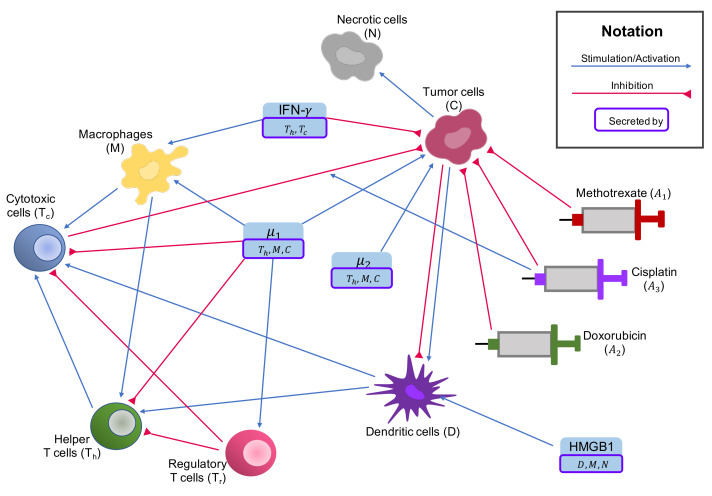
**Interaction network with chemotherapy drugs.** Activation effects, proliferation effects, and stimulation effects are indicated by blue arrows; and inhibitory effects are indicated by red arrows. Chemotherapy drugs also inhibit all immune cells (red arrows from drugs to immune cells not shown).

We build upon this system of ODE by adding the interactions of the variables in this system with the following chemotherapy drugs: methotrexate, doxorubicin, and cisplatin. The interaction network of these drugs with cells and cytokines of osteosarcoma tumor microenvironment is shown in Figure 1. We used an exponential kill model, as introduced in [84], to describe how chemotherapy affects the cancer microenvironment, and modeled the change in population of the new model’s variables over time in the unit of days. The details of the effects of chemotherapy drugs on immune cells and cancer cells are explained below (changes to Equations (1)–(14) are in bold).

#### 2.1.1. Cancer Cells

All chemotherapy drugs in our model aim to kill tumor cells, though they have different mechanisms of action. Methotrexate hinders DNA synthesis in fast dividing cancer cells by inhibiting folate-dependent pathways [85]. Doxorubicin can kill cancer cells by binding to DNA-associated enzymes, intercalating the base pair of DNA’s double helix, and targeting many molecular targets such as topoisomerase enzymes I and II, which results in DNA damage [86]. Cisplatin binds platinum to DNA by forming inter-stranded and intra-stranded crosslinks, and thus induces DNA damage which leads to cell death in rapidly proliferating cells [87,88].

Similarly to [84,89], we use saturation kill term $\left(1-e^{\beta A}\right)$ to model the direct cytotoxic effects of chemotherapy drugs on cancer cells, where β is the drug efficacy parameter, and *A* is the drug concentration at the tumor site. This is based on the observation that at low concentrations, the cancer killing effects of these drugs are almost linear, but at very high concentrations the cancer killing effects plateau. Unlike doxorubicin and cisplatin, methotrexate can only eliminate cancer cells during certain phases of the cell cycle, so we added the term $\left(f-\frac{\tau }{a}+\frac{1}{24a}\right)$ to methotrexate’s cytotoxic effect to account for this phenomenon, as modeled in [84]. Here, *f* denotes the fraction of cells in the vulnerable phase of the cell cycle for methotrexate, *a* denotes cell cycle time in days, and τ is defined to be minimum(T,fa), with *T* being drug exposure time in days.

Besides its direct role in targeting tumor cells, cisplatin has also been reported to increase cytotoxic cells’ cancer killing capability by upregulating MHC-1 expression in cancer cells [35,87,90,91]. We also use a saturation term to describe this effect, as it is very likely that a high concentration of cisplatin can also plateaus in the upregulation of MHC-1 in cancer cells. We make the assumption that the concentration of cisplatin at which this effect slows down is about the same concentration at which the cancer killing effect of cisplatin slows down, so we use the same drug efficacy parameter $\beta_{3}$ in both terms, resulting in the following equation for cancer cells:

(15) $$\begin{array}{cc} \frac{d\left[C\right]}{dt}=\; & \left(\lambda_{C}+\lambda_{C\mu_{1}}\left[\mu_{1}\right]+\lambda_{C\mu_{2}}\left[\mu_{2}\right]\right)\left[C\right]\left(1-\frac{\left[C\right]}{C_{0}}\right)\\ \; & -(\delta_{CI_{\gamma }}\left[I_{\gamma }\right]+\delta_{C}+\delta_{CT_{c}}\left(1+\delta_{CT_{c}A_{3}}\left(1-e^{-\beta_{3}A_{3}}\right)\right)\left[T_{c}\right])\left[C\right]\\ \; & -(K_{C}\left(f-\frac{\tau }{a}+\frac{1}{24a}\right)\left(1-e^{-\beta_{1}A_{1}}\right)+K_{C}\left(1-e^{-\beta_{2}A_{2}}\right)\\ \; & +K_{C}\left(1-e^{-\beta_{3}A_{3}}\right))\left[C\right] \end{array}$$

where $\delta_{CT_{c}A_{3}}$ represents cisplatin’s promotion of cytotoxic cells’ cancer killing ability; $K_{C}$ is rate of chemotherapy-induced tumor death; and $\beta_{1}$, $\beta_{2}$, and $\beta_{3}$ are the medicine efficacy coefficients of methotrexate, doxorubicin, and cisplatin, respectively. A description of every chemotherapy-related parameter in our model is given in Table 2.

#### 2.1.2. Necrotic Cells

As a proportion of cancer cells killed by chemotherapy drugs become necrotic cells, we describe the change in population of necrotic cells with the presence of chemotherapy as follows:

(16) $$\begin{array}{cc} \frac{d\left[N\right]}{dt}=\; & \alpha_{NC}(\delta_{CI_{\gamma }}\left[I_{\gamma }\right]+\delta_{C}+\delta_{CT_{c}}\left(1+\delta_{CT_{c}A_{3}}\left(1-e^{-\beta_{3}A_{3}}\right)\right)\left[T_{c}\right])\left[C\right]\\ \; & +\alpha_{\mathrm{NCA}}(K_{C}\left(f-\frac{\tau }{a}+\frac{1}{24a}\right)\left(1-e^{-\beta_{1}A_{1}}\right)+K_{C}\left(1-e^{-\beta_{2}A_{2}}\right)\\ \; & +K_{C}\left(1-e^{-\beta_{3}A_{3}}\right))\left[C\right]-\delta_{N}\left[N\right] \end{array}$$

where $\alpha_{NCA}$ is the fraction of dying cancer cells induced by chemotherapy that turn into necrotic cells.

#### 2.1.3. Immune Cells

Since chemotherapy does not only eliminate tumor cells but also kills immune cells, we include the effects of chemotherapy in the equations of immune cells as well. Similarly to [89], we assume that the same quantity of chemotherapy drugs is required to affect cancer cells and immune cells, even when the rate at which chemotherapy kills cancer cells is different than the rate at which it kills immune cells. Hence, we use the same drug efficacy coefficients for cancer and immune cells, but different rates of drug-induced cell death, leading to the following modified immune cell equations:

(17) $$\begin{array}{cc} \frac{d\left[M_{N}\right]}{dt}=\; & A_{M_{N}}-\left(\lambda_{MI_{\gamma }}\left[I_{\gamma }\right]+\lambda_{M\mu_{1}}\left[\mu_{1}\right]\right)\left[M_{N}\right]-\delta_{M_{N}}\left[M_{N}\right]\\ \; & -(K_{M_{N}}\left(f-\frac{\tau }{a}+\frac{1}{24a}\right)\left(1-e^{-\beta_{1}A_{1}}\right)+K_{M_{N}}\left(1-e^{-\beta_{2}A_{2}}\right)\\ \; & +K_{M_{N}}\left(1-e^{-\beta_{3}A_{3}}\right))\left[M_{N}\right] \end{array}$$

(18) $$\begin{array}{cc} \frac{d\left[M\right]}{dt}=\; & \left(\lambda_{MI_{\gamma }}\left[I_{\gamma }\right]+\lambda_{M\mu_{1}}\left[\mu_{1}\right]\right)\left[M_{N}\right]-\delta_{M}\left[M\right]-(K_{M}\left(f-\frac{\tau }{a}+\frac{1}{24a}\right)\left(1-e^{-\beta_{1}A_{1}}\right)\\ \; & +K_{M}\left(1-e^{-\beta_{2}A_{2}}\right)+K_{M}\left(1-e^{-\beta_{3}A_{3}}\right))\left[M\right] \end{array}$$

(19) $$\begin{array}{cc} \frac{d\left[T_{N}\right]}{dt}=\; & A_{T_{N}}-\left(\lambda_{T_{h}M}\left[M\right]+\lambda_{T_{h}D}\left[D\right]\right)\left[T_{N}\right]-\lambda_{T_{r}\mu_{1}}\left[\mu_{1}\right]\left[T_{N}\right]\\ \; & -\left(\lambda_{T_{c}T_{h}}\left[T_{h}\right]+\lambda_{T_{c}M}\left[M\right]+\lambda_{T_{c}D}\left[D\right]\right)\left[T_{N}\right]-\delta_{T_{N}}\left[T_{N}\right]\\ \; & -(K_{T_{N}}\left(f-\frac{\tau }{a}+\frac{1}{24a}\right)\left(1-e^{-\beta_{1}A_{1}}\right)+K_{T_{N}}\left(1-e^{-\beta_{2}A_{2}}\right)\\ \; & +K_{T_{N}}\left(1-e^{-\beta_{3}A_{3}}\right))\left[T_{N}\right] \end{array}$$

(20) $$\begin{array}{cc} \frac{d\left[T_{h}\right]}{dt}=\; & \left(\lambda_{T_{h}M}\left[M\right]+\lambda_{T_{h}D}\left[D\right]\right)\left[T_{N}\right]-\left(\delta_{T_{h}T_{r}}\left[T_{r}\right]+\delta_{T_{h}\mu_{1}}\left[\mu_{1}\right]+\delta_{T_{h}}\right)\left[T_{h}\right]\\ \; & -(K_{T_{h}}\left(f-\frac{\tau }{a}+\frac{1}{24a}\right)\left(1-e^{-\beta_{1}A_{1}}\right)+K_{T_{h}}\left(1-e^{-\beta_{2}A_{2}}\right)\\ \; & +K_{T_{h}}\left(1-e^{-\beta_{3}A_{3}}\right))\left[T_{h}\right] \end{array}$$

(21) $$\begin{array}{cc} \frac{d\left[T_{r}\right]}{dt}=\; & \left(\lambda_{T_{r}\mu_{1}}\left[\mu_{1}\right]\right)\left[T_{N}\right]-\delta_{T_{r}}\left[T_{r}\right]-(K_{T_{r}}\left(f-\frac{\tau }{a}+\frac{1}{24a}\right)\left(1-e^{-\beta_{1}A_{1}}\right)\\ \; & +K_{T_{r}}\left(1-e^{-\beta_{2}A_{2}}\right)+K_{T_{r}}\left(1-e^{-\beta_{3}A_{3}}\right))\left[T_{r}\right] \end{array}$$

(22) $$\begin{array}{cc} \frac{d\left[T_{c}\right]}{dt}=\; & \left(\lambda_{T_{c}T_{h}}\left[T_{h}\right]+\lambda_{T_{c}M}\left[M\right]+\lambda_{T_{c}D}\left[D\right]\right)\left[T_{N}\right]-\left(\delta_{T_{c}T_{r}}\left[T_{r}\right]+\delta_{T_{c}\mu_{1}}\left[\mu_{1}\right]+\delta_{T_{c}}\right)\left[T_{c}\right]\\ \; & -(K_{T_{c}}\left(f-\frac{\tau }{a}+\frac{1}{24a}\right)\left(1-e^{-\beta_{1}A_{1}}\right)+K_{T_{c}}\left(1-e^{-\beta_{2}A_{2}}\right)\\ \; & +K_{T_{c}}\left(1-e^{-\beta_{3}A_{3}}\right))\left[T_{c}\right] \end{array}$$

(23) $$\begin{array}{cc} \frac{d\left[D_{N}\right]}{dt}=\; & A_{D_{N}}-\left(\lambda_{DC}\left[C\right]+\lambda_{DH}\left[H\right]\right)\left[D_{N}\right]-\delta_{D_{N}}\left[D_{N}\right]\\ \; & -(K_{D_{N}}\left(f-\frac{\tau }{a}+\frac{1}{24a}\right)\left(1-e^{-\beta_{1}A_{1}}\right)+K_{D_{N}}\left(1-e^{-\beta_{2}A_{2}}\right)\\ \; & +K_{D_{N}}\left(1-e^{-\beta_{3}A_{3}}\right))\left[D_{N}\right] \end{array}$$

(24) $$\begin{array}{cc} \frac{d\left[D\right]}{dt}=\; & \left(\lambda_{DC}\left[C\right]+\lambda_{DH}\left[H\right]\right)\left[D_{N}\right]-\left(\delta_{DC}\left[C\right]+\delta_{D}\right)\left[D\right]\\ \; & -(K_{D}\left(f-\frac{\tau }{a}+\frac{1}{24a}\right)\left(1-e^{-\beta_{1}A_{1}}\right)+K_{D}\left(1-e^{-\beta_{2}A_{2}}\right)\\ \; & +K_{D}\left(1-e^{-\beta_{3}A_{3}}\right))\left[D\right] \end{array}$$

where $K_{M_{N}}$, $K_{M}$, $K_{T_{N}}$, $K_{T_{h}}$, $K_{T_{r}}$, $K_{T_{c}}$, $K_{D_{N}}$, and $K_{D}$ are the rates of chemotherapy-induced cell death of naive macrophages, macrophages, naive T cells, helper T cells, regulatory T cells, cytotoxic cells, naive dendritic cells, and dendritic cells, respectively.

#### 2.1.4. Chemotherapy Drugs

Chemotherapy drugs are given through IV infusion in osteosarcoma treatments, so their bioavailability is 100%. Thus, we use the following equations to model the changes in concentration of the chemotherapy drugs at the tumor site over time:

(25) $$\begin{array}{cc} \frac{d[A_{1}]}{dt}=\; & v_{A_{1}}\left(t\right)-\delta_{A_{1}}\left[A_{1}\right] \end{array}$$

(26) $$\begin{array}{cc} \frac{d[A_{2}]}{dt}=\; & v_{A_{2}}\left(t\right)-\delta_{A_{2}}\left[A_{2}\right] \end{array}$$

(27) $$\begin{array}{cc} \frac{d[A_{3}]}{dt}=\; & v_{A_{3}}\left(t\right)-\delta_{A_{3}}\left[A_{3}\right] \end{array}$$

Here, $v_{A_{1}}\left(t\right)$, $v_{A_{2}}\left(t\right)$, and $v_{A_{3}}\left(t\right)$ are the amounts of methotrexate, doxorubicin, and cisplatin injected per day per liter of body volume, with the unit of mg/L per day; $\delta_{A_{1}}$, $\delta_{A_{2}}$, and $\delta_{A_{3}}$ are the decay rates of methotrexate, doxorubicin, and cisplatin, respectively.

### 2.2. Data of the Model

#### 2.2.1. Tumor Microenvironment Data

**Figure 2:**
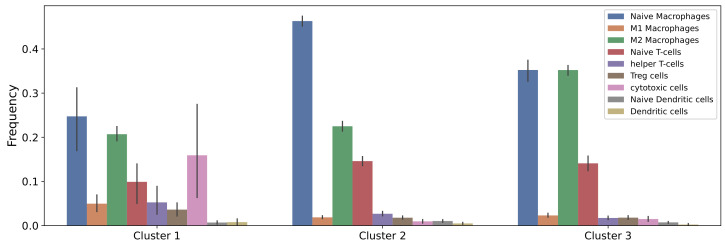
**Estimated immune infiltrates in each cluster.** Clusters were derived according to variations in 22 immune cell types of osteosarcoma tumors.

A recent study showed that among the available digital cytometry methods with which to deconvolve the gene expression data of a tissue into relative abundances of cells in the tissue, CIBERSORTx B-mode performs best [92]. In our previous study [58], we used the gene expression data of 88 osteosarcoma tumors from TARGET data set and applied CIBERSORTx B-mode to estimate the fractions of immune cells in each of these tumors. Then, k-means clustering was performed on the estimated cell fractions, and three clusters of osteosarcoma tumors with distinctive immune patterns were found. In this work, we study the impacts of chemotherapy drugs on the same three clusters. The average immune fractions in each cluster are presented in Figure 2.

To obtain immune cell populations, we multiplied the immune fractions estimated from CIBERSORTx by a scaling factor $\alpha_{dim}$. From the supplementary data of TARGET project, which include information on the percentage of tumor, necrotic, stroma, and normal cells of each sample, we derived the cancer and necrotic populations from immune populations. We used the percentage of normal cells to denote the percentage of total immune cells. In particular, given total immune population *I*, we computed the populations of cancer and necrotic cells as follows:

(28) $$C=I\times \frac{\%\mathrm{of}\;\mathrm{cancer}\;\mathrm{cells}}{\%\mathrm{of}\;\mathrm{total}\;\mathrm{immune}\;\mathrm{cells}},$$

(29) $$N=I\times \frac{\%\mathrm{of}\;\mathrm{necrotic}\;\mathrm{cells}}{\%\mathrm{of}\;\mathrm{total}\;\mathrm{immune}\;\mathrm{cells}},$$

where *C* and *N* are the populations of cancer and necrotic cells, respectively.

We chose $\alpha_{dim}$ based on the average cancer cell population of osteosarcoma. The mean volume of Ewing sarcomas is reported to be 275 mL [93], and a study found Ewing sarcoma volumes to be similar to osteosarcoma volumes [94]. Therefore, we approximated the mean volume of osteosarcoma to be 275 mL. Since osteoblasts have a diameter of 20–50 μm [95], we estimated that osteosarcoma cells, which are malignant osteoblasts, have an average diameter of 35 μm, leading to an estimated average of $6.4\times 10^{9}$ cancer cells in osteosarcoma tumors. Thus, $\alpha_{dim}$ was chosen to be $1.765\times 10^{8}$ to match the average cancer cell population among all patients in TARGET data to $6.4\times 10^{9}$. It is worth noting that a smaller size of osteoblasts has also been reported [96]. However, $\alpha_{dim}$ is simply a scaling factor and has no impacts on the dynamics of cells and cytokines in our system; thus, the size of osteoblasts does not affect our results.

#### 2.2.2. Treatment Data

Given a treatment regimen of interest, we applied its standard dosage to our model. Most doses of chemotherapy drugs for osteosarcoma are measured in mg/m${}^{2}$, but we modeled the drug concentration at the tumor site in milligrams per liter of body volume. We therefore need to convert drug doses from mg/m${}^{2}$ to mg/L. We used an average body surface area of a human male of 1.9 m${}^{2}$[97] and an average male body volume of 59.7 L [98] for conversion. That is, for example, 75 mg/m${}^{2}$ would be equivalent to:

(30) $$\begin{array}{c} 75\;{\mathrm{mg}/m}^{2}=\frac{75\;\mathrm{mg}}{m^{2}}\times \frac{1.9\;m^{2}}{59.7\;L}=2.3869\;\mathrm{mg}/L \end{array}$$

### 2.3. Parameter Values

The drug efficacy coefficients, cell cycle time, and fraction of cells in the vulnerable phase of the cell cycle are taken from [84]. Using the molecular mass of chemotherapy drugs [89,99,100,101], we can convert the drug efficacy coefficients given in [84] to units of (mg/L)${}^{-1}$:

(31) $$\begin{array}{cc} \beta_{1}= & \;\left(\frac{1.126\;L}{\mu \mathrm{mol}}\right)\left(\frac{10^{6}\;\mu \mathrm{mol}}{1\;\mathrm{mol}}\right)\left(\frac{1\;\mathrm{mol}}{454.4\;g\;\mathrm{MTX}}\right)\left(\frac{1\;g}{1000\;\mathrm{mg}}\right)=2.4780\;L/\mathrm{mg} \end{array}$$

(32) $$\begin{array}{cc} \beta_{2}= & \;\left(\frac{1.063\;L}{\mu \mathrm{mol}}\right)\left(\frac{10^{6}\;\mu \mathrm{mol}}{1\;\mathrm{mol}}\right)\left(\frac{1\;\mathrm{mol}}{580\;g\;\mathrm{DOX}}\right)\left(\frac{1\;g}{1000\;\mathrm{mg}}\right)=1.8328\;L/\mathrm{mg} \end{array}$$

(33) $$\begin{array}{cc} \beta_{3}= & \;\left(\frac{0.044\;L}{\mu \mathrm{mol}}\right)\left(\frac{10^{6}\;\mu \mathrm{mol}}{1\;\mathrm{mol}}\right)\left(\frac{1\;\mathrm{mol}}{300\;g\;\mathrm{CDDP}}\right)\left(\frac{1\;g}{1000\;\mathrm{mg}}\right)=0.1467\;L/\mathrm{mg} \end{array}$$

Drug efficacy coefficients for doxorubicin-resistant and cisplatin-resistant cells were also included in [84], and can be converted in a similar way. The values for all chemotherapy-related parameters in our model and their sources are given in Table 2.

The fraction of cancer cells killed by chemotherapy, $K_{C}$, was taken from [89,102] to be 0.9, based on the notion that chemotherapy strength is one log kill [103]. Since chemotherapy is more effective at eliminating fast proliferating cells, it is safe to assume that the rates of chemotherapy-induced death of immune cells are smaller than those of chemotherapy-induced death of cancer cells. Hence, $K_{M_{N}}$, $K_{M}$, $K_{T_{N}}$, $K_{T_{h}}$, $K_{T_{r}}$, $K_{T_{c}}$, $K_{D_{N}}$, and $K_{D}$ are assumed to be smaller but in the same order of magnitude as $K_{C}$, and we use a value of 0.6 for all of them, as in [102]. Decay rates of chemotherapy drugs were derived from their elimination half lives in the following way:

(34) $$\begin{array}{c} \delta_{A}=\frac{ln2}{\mathrm{half}\;\mathrm{life}\;\mathrm{of}\;A\;\mathrm{in}\;\mathrm{days}} \end{array}$$

where $\delta_{A}$ is the decay rate of *A*. On average, the elimination half lives of doxorubicin, cisplatin, and high-dose methotrexate are 2 h, 25 min, and 11.5 h respectively [104,105,106], resulting in the corresponding decay rates of 8.3178, 39.9253, and 1.4466.

As no values for $\alpha_{NCA}$ and $\delta_{CT_{c}A_{3}}$ can be found in the literature, we assume biologically reasonable values for these parameters. $\alpha_{NCA}$ is the fraction of dying cancer cells induced by chemotherapy that become necrotic cells, so it is bounded between 0 and 1. We make the assumption that a large proportion of dying cancer cells from treatment turn into necrotic cells, and set $\alpha_{NCA}$ = 0.8. For $\delta_{CT_{c}A_{3}}$, we assume that cisplatin at maximum effect can double the cancer killing ability of cytotoxic cells, or equivalently $\delta_{CT_{c}A_{3}}$ = 1. In order to investigate whether our assumptions for those parameters have large impacts on the cancer cell population, we performed sensitivity analysis and studied the change in cancer cell population after treatment while varying these two parameters.

### 2.4. Non-Dimensionalization

To achieve additional numerical stability, non-dimensionalization of the whole system was carried out. For each variable *X* of the original system in [58], its dimensionless form can be written as:

(35) $$\begin{array}{c} \bar{X}=\frac{X}{X^{\infty }}, \end{array}$$

where $X^{\infty }$ is the steady state value of *X* given in [58]. For the newly added variables, which are the chemotherapy drugs, we introduced the following non-dimensional variables:

(36) $$\begin{array}{c} \bar{A}=\frac{A\;\delta_{A}}{v_{A}^{*}}, \end{array}$$

where *A* is the dimensional variable, $\delta_{A}$ is the decay rate of *A*, and $v_{A}^{*}$ is the amount of drug *A* injected on its first injection day of the treatment. Further details on non-dimensionalization are given in Appendix A.

To solve the non-dimensional system of ordinary differential equations, we used the solve_ivp function from Scipy package in python [107], with initial conditions from a chosen data point of interest in each cluster.

### 2.5. Sensitivity Analysis

We performed local gradient-based sensitivity analysis on all chemotherapy-related parameters to study their impacts on the outputs of the system. For the non-dimensional system $\frac{d\bar{X}}{dt}=F\left(\bar{X},\theta ,t\right)$ with model parameters $\theta =\theta_{1},...,\theta_{N}$, the local (first order) sensitivity of parameter $\theta_{i}$ with respect to the variable *X* is defined as [108]:

(37) $$\begin{array}{c} s_{i}=\frac{\partial \bar{X}}{\partial \theta_{i}} \end{array}$$

As we are mainly interested in how drug-related parameters affect the number of cancer cells, we calculated the sensitivity of treatment parameters with respect to cancer and total cell populations. Since the effects of the treatment do not reach steady state, we consider time-dependent sensitivity. That is, we measure sensitivity of parameters in every time step throughout the treatment and some time after. The change in sensitivity of $\theta_{i}$ over time can be derived as follows:

(38) $$\begin{array}{c} \frac{\partial s_{i}}{\partial t}=\frac{\partial }{\partial t}\left(\frac{\partial \bar{X}}{\partial \theta_{i}}\right)=\frac{\partial }{\partial \theta_{i}}\left(\frac{\partial \bar{X}}{\partial t}\right)=\frac{\partial F(\bar{X},\theta_{i},t)}{\partial \theta_{i}} \end{array}$$

By applying the chain rule, we have:

(39) $$\begin{array}{c} \frac{\partial s_{i}}{\partial t}=\frac{\partial F}{\partial \theta_{i}}+\frac{\partial F}{\partial \bar{X}}s_{i} \end{array}$$

In addition, we also look at the relative sensitivity, which is commonly used in metabolic control analysis of biological networks [108]:

(40) $$\begin{array}{c} {\bar{s}}_{i}\left(t\right)=s_{i}\left(t\right)\frac{\theta_{i}}{\bar{X}\left(t\right)} \end{array}$$

Then, we compute the average sensitivity of each type over a period of time T:

(41) $$\begin{array}{cccc} S_{i}= & \;\frac{1}{T}\int_{0}^{T}s_{i}\left(t\right)dt,\; & {\bar{S}}_{i}= & \;\frac{1}{T}\int_{0}^{T}{\bar{s}}_{i}\left(t\right)dt \end{array}$$

The sensitivity varies for different values of the parameters, so we consider a small neighborhood Ω(θ) of the given parameter set and calculate:

(42) $$\begin{array}{cccc} S_{i}= & \;\int_{\Omega }S_{i}\left(\theta \right)d\theta ,\; & {\bar{S}}_{i}= & \;\int_{\Omega }{\bar{S}}_{i}\left(\theta \right)d\theta \end{array}$$

where the integrals are computed using a numerical technique called sparse grid points [109,110]. In particular, to evaluate $S_{i}$, sparse grid point method samples $\theta_{k}$ from parameter space Ω for k=1,…,m, where the number of samples *m* are chosen by the user. Then $S_{i}\left(\theta_{k}\right)$ is evaluated for k=1,…,m, and the summation $\sum_{k=1}^{m}S_{i}\left(\theta_{k}\right)$ is used as an approximation to $S_{i}$. Here, to generate sparse grid points samples, we apply Matlab package *nwspgr* provided by [109], and we use m=39.

### 2.6. Optimization of Drug Dosage

We introduce a framework to find the appropriate dose of a given treatment regimen for each patient. With it, one aims to achieve a target cancer cell population at a given time *t*, typically to reduce the size of tumor for the day of surgery. Hence, we want to minimize the difference between the estimated cancer cell population at *t* from our model and the target cancer cell population. The least square error is used to model this difference, as least square error penalizes high errors more than absolute error, and is less computationally expensive than hinge loss.

Since high doses of chemotherapy are known to induce high toxicity to the patient, we would like to achieve cancer cell populations close to the target population with the smallest dosages possible. Therefore, we added a L1 norm regularization term, which is the sum of absolute values of drug dosages, to the loss function. We used the L1 norm instead of the L2 norm, because the L2 norm penalizes high doses of individual drugs more severely. In most osteosarcoma treatments, methotrexate is used at a much higher dose than other drugs, so L2 norm would put too much weight on methotrexate dose and neglect other drugs’ doses.

As patients can only tolerate certain dosages of chemotherapy and drug doses cannot be negative, we also put lower and upper bounds on the drug doses. Thus, we have the following loss function:

(43) $$\begin{array}{c} L\left(v,t\right)={\left(\hat{C}\left(v,t\right)-C_{target}\right)}^{2}+\kappa \sum_{i=1}^{M}\left|v_{i}\right|\\ \mathrm{subject}\;\mathrm{to}\;\;0\leq v_{i}\leq U_{i},i=1,\ldots ,M \end{array}$$

Here, *v* is a vector of length *M*, denoting the doses of the *M* drugs in the given treatment. *t* is the time of interest at which cancer cell population is evaluated for optimization. $\hat{C}\left(v,t\right)$ is the cancer cell population with drug doses *v* at time *t* of interest, and is computed via our ODE solver. $C_{target}$ is the target cancer cell population at time *t*, and is chosen by the user. $U_{i}$ is the highest allowable value for $v_{i}$. κ is the regularization parameter; the higher κ is, the more the optimizer focuses on achieving small doses and less on achieving small error between $\hat{C}\left(v,t\right)$ and $C_{target}$.

The optimize.minimize function from Scipy package in python is used to solve this optimization problem, with the outputs being the optimal doses.

## 3. Results

### 3.1. Dynamics of the Cancer Microenvironment with MAP Treatment

Typical treatments for osteosarcoma include neoadjuvant chemotherapy, usually for 10 weeks, then surgery, and adjuvant chemotherapy after the surgery for up to a year [40]. The most common chemotherapy regimen for osteosarcoma in children and young adults is the MAP regimen, which is a combination of doxorubicin, cisplatin, and high-dose methotrexate [39]. This regimen consists of six 35-day cycles; two cycles are applied before surgery and the remaining four are applied after surgery. In each cycle, 37.5 mg/m${}^{2}$ of doxorubicin and 60 mg/m${}^{2}$ of cisplatin are administered through IV per day on days 1 and 2, and 12,000 mg/m${}^{2}$ of methotrexate is administered through IV over 4 h per day on days 22 and 29 [111,112]. Different infusion schedules have been used for doxorubicin and cisplatin: doxorubicin can be injected as a bolus or a 4-h infusion each day, or a continuous infusion over 48 h, whereas cisplatin can be injected over 2 or 4 h each day, or continuously over 72 h [112]. We studied the dynamics of cell and cytokine populations in large osteosarcoma tumors during neoadjuvant MAP treatment, which includes two 35-day cycles. In particular, we used the steady state values of cells and cytokines in [58] as initial conditions, the typical dosage of the MAP regimen as the drug inputs, and 4-h infusions on previously specified days as the injection schedule for each drug. We set the start of chemotherapy treatment to be 7 days after biopsy, as it usually takes a few days to receive the results of the biopsy.

**Figure 3:**
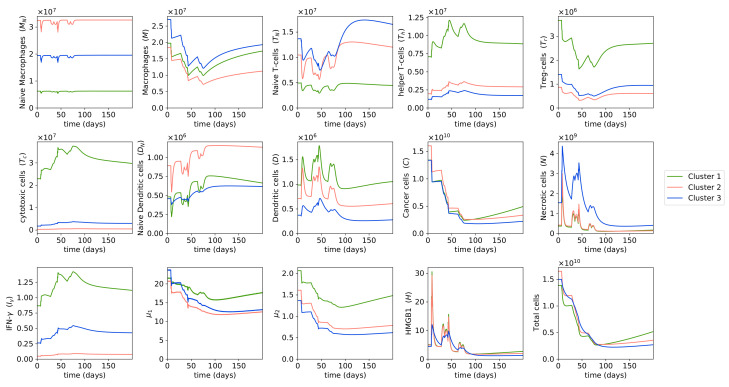
**Dynamics with MAP treatment.** Behaviors of cells and cytokines in osteosarcoma tumors during the MAP treatment and a few months after treatment. Initial conditions are large tumors in each cluster, i.e., the without-treatment steady state values of each cluster. Drug doses are the typical doses of the MAP regimen. The different color lines indicate the dynamics of different clusters.

Figure 3 shows that for all clusters, cancer cell populations are reduced significantly after two cycles of MAP treatment. It is important to note that the cancer cell populations do not reach zero after chemotherapy, so cancer cells will start growing again after chemotherapy. However, the goal of neoadjuvant therapy is not to eradicate cancer cells completely, but only to reduce the boundaries of the tumor and to remove any small metastases that have not been detected [113].

Cluster 2 had the highest cancer cell population at the start of treatment, so naturally cluster 2 also had the highest cancer cell population left after neoadjuvant therapy. Interestingly, cluster 1 had approximately the same number of cancer cells as cluster 3 at the start of treatment, but ended up with a higher cancer cell population than cluster 3 after treatment. This is because in each chemotherapy cycle, there are a few weeks where no chemotherapy drugs are administered in order to allow the patient to recover from the drugs’ toxicity, and thus during these few weeks, cancer cells can start growing again. Cluster 1’s cancer cell population, which was reported in [58] to have the highest growth rate in the three clusters, grew more during the weeks with no drugs given, resulting in a higher number of cancer cells after treatment compared to cluster 3. This observation suggests that we should take the patient’s cancer growth rate into account when choosing their chemotherapy dosage.

During the MAP treatment, necrotic cells, dendritic cells, and HMGB1 oscillated between increasing and decreasing in abundance. Since chemotherapy drugs aim to kill cancer cells, and a fraction of dying cancer cells become necrotic cells, the population of necrotic cells increases on the days the drugs are injected and there are many drug-induced dying cancer cells. However, during the weeks where no chemotherapy drugs are administered, only drug-free dying cancer cells can become necrotic cells, and with the cancer cell population being already reduced by the previously given drugs, the number of drug-free dying cancer cells is small, leading to a decrease in necrotic cell population.

HMGB1 is mainly produced by necrotic cells, so HMGB1 abundance increases when the necrotic population increases and decreases when the necrotic population decreases. Meanwhile, dendritic cells are activated largely by HMGB1, so the dynamics of dendritic cells share the same trend as the dynamics of HMGB1. That means both HMGB1 and dendritic cells increase on the days chemotherapy drugs are administered and decrease on the weeks with no drugs given. An increase in dendritic cell population right after doxorubicin [114,115,116,117] or cisplatin [35,36,87] administration, and a rise in HMGB1 production following doxorubicin [116,117,118], have been shown in several studies, which aligns with our results.

We observed that in general, helper T cells, cytotoxic cells, and IFN-γ increase in population during chemotherapy. There are many studies that report an increase in helper T cells’ and/or cytotoxic T cells’ abundance due to doxorubicin [117,118,119,120,121,122], cisplatin [35,87,90,123] or methotrexate [124], and thus they support our findings. Doxorubicin has been known to induce immunogenic cell death, which leads to the maturation of dendritic cells and accordingly the activation of helper and cytotoxic T cells [114,120,125]. IFN-γ is produced by helper T cells and cytotoxic cells; thus, IFN-γ abundance also increases as these two cells increase in population during MAP treatment. The increase in IFN-γ level after administration of doxorubicin and cisplatin has also been observed in multiple studies [87,116,117,126].

On the other hand, populations of macrophages, regulatory T cells, cytokines groups $\mu_{1}$, and $\mu_{2}$ mainly decrease during MAP treatment. These immune cells are not affected by the necrotic cell death process caused by chemotherapy, so they decrease in population during chemotherapy, as they are also killed by the drugs. $\mu_{1}$ and $\mu_{2}$ are produced by helper T cells, macrophages, and cancer cells. Even though the helper T cell population increases during treatment, macrophage and cancer cell populations decrease more so, which leads to an overall decrease in $\mu_{1}$ and $\mu_{2}$ throughout MAP treatment. Several other studies have also reported a reduction in regulatory T cell quantity due to cisplatin [35,87,90] and a decrease in the level of IL-6, which is the main component of $\mu_{2}$, due to methotrexate and doxorubicin [124,126,127,128].

### 3.2. Sensitivity Analysis

**Figure 4:**
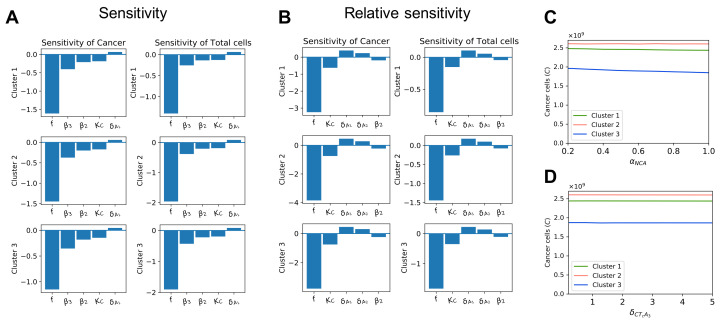
**Sensitivity of chemotherapy-related parameters.** Sub-figure (**A**) shows the local sensitivity of the 5 most sensitive treatment-related parameters with respect to cancer cell population and total cell population. Sub-figure (**B**) shows the local relative sensitivity of the 5 most sensitive treatment-related parameters with respect to cancer cell population and total cell population. Sub-figures (**C**,**D**) display the cancer cell population after treatment with different values of $\alpha_{NCA}$ and $\delta_{CT_{c}A_{3}}$, respectively.

To study the impacts of the newly introduced parameters on the outputs of the model, we performed local sensitivity analysis on the chemotherapy-related parameters using the non-dimensional system. The initial conditions for sensitivity analysis are the large tumors in each cluster, which we used without-treatment steady state values to represent. It is worth noting that the cell cycle time, *a*, was not included in this sensitivity analysis, because it is a simple measurement rather than a parameter that needs to be estimated or fitted to the experimental data. The most sensitive time-averaged parameters in terms of sensitivity and relative sensitivity are presented in Figure 4.

In all clusters, the initial fraction of cells in the vulnerable phase of the cell cycle *f* has the largest impact on cancer cell population among treatment-related parameters according to both the sensitivity and relative sensitivity analyses. The rate of chemotherapy-induced cancer cell death, $K_{C}$, and the drug efficacy coefficients of doxorubicin and cisplatin, $\beta_{2}$ and $\beta_{3}$, are also sensitive to cancer and total cell population during treatment. Meanwhile, the drug efficacy coefficient of methotrexate, $\beta_{1}$, does not seem as sensitive, but the decay rate of methotrexate is.

We notice that the parameters whose values are assumed, $\alpha_{NCA}$ and $\delta_{CT_{c}A_{3}}$, do not have large effects on the cancer cell population or total cell population based on the results of the sensitivity analysis. To further confirm this, we also plotted the cancer cell populations after treatment with different values of these parameters. We chose $\alpha_{NCA}$ ranging from 0.2 to 1, because it is a fraction and thus is bounded between 0 and 1, and $\delta_{CT_{c}A_{3}}$ ranges from 0.2 to 5 times its original value. Figure 4C,D shows that varying either of these parameters results in negligible changes to cancer cell population after treatment.

### 3.3. Dynamics of the Cancer Microenvironment in Chemo-Resistant Tumors with MAP Treatment

The effectiveness of chemotherapy is highly dependent on the existence of resistant cancer cells. We are interested in studying the changes in quantity of cells and cytokines when osteosarcoma cells are resistant to one or multiple drugs within the MAP regimen. As mentioned in Section 2, we obtained drug efficacy coefficients from [84]; these values were estimated to fit the survival data of cancer cells under different chemotherapy drugs. The same study [84] also included the estimated drug efficacy coefficients of doxorubicin and cisplatin in doxorubicin-resistant and cisplatin-resistant cancer cells, respectively. Using these parameter values, we plotted the dynamics in the osteosarcoma microenvironment during MAP treatment when cancer cells are resistant to either doxorubicin or cisplatin, or to both drugs. Since methotrexate-resistant cells were not used in [84], and hence no parameter values were available for them, we did not model the dynamics with methotrexate-resistant cells.

**Figure 5:**
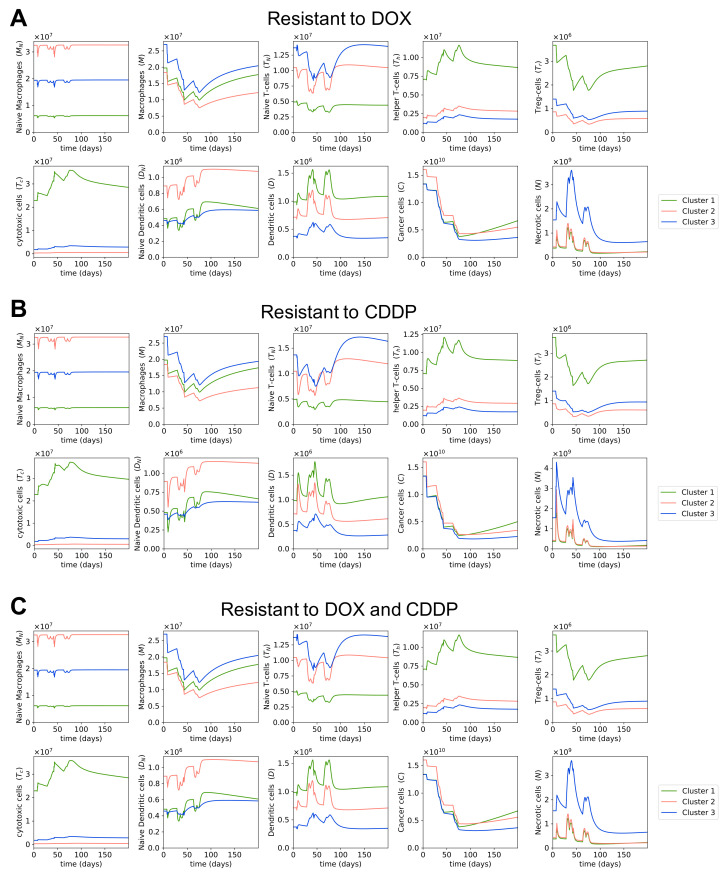
**Dynamics in chemotherapy-resistant cells with MAP treatment.** Sub-figures (**A**–**C**) show the dynamics of immune, cancer, and necrotic cells in osteosarcoma during the MAP treatment and a few months after treatment when cancer cells are resistant to doxorubicin, cisplatin, and both doxorubicin and cisplatin, respectively.

Figure 5A shows that MAP treatment is not as effective at shrinking the tumor when cancer cells are resistant to doxorubicin; the cancer cell population after treatment is about 60% to 70% higher in doxorubicin-resistant cells than in non-doxorubicin-resistant cells (Table 3). The smaller reduction in cancer cell population of doxorubicin-resistant cells during doxorubicin administration means fewer necrotic cells are produced in the process, and accordingly a lower level of dendritic cells (Figure 5A), as necrotic cells indirectly promote dendritic cell maturation through the release of HMGB1. We notice no clear difference in the dynamics of T cells and macrophages compared to the microenvironment of non-doxorubicin-resistant cells. It is worth noting that we modeled only cancer cells to be resistant to chemotherapy drugs, so immune cells were by design not resistant to these drugs.

On the other hand, with cisplatin-resistant cells, we observed little difference in the reduction of cancer cell population compared to non-cisplatin-resistant cells (Figure 5B, Table 3). This is due to the fact that cisplatin’s drug efficacy parameter, $\beta_{3}$, is small compared to methotrexate and doxorubicin’s drug efficacy parameters, resulting in cisplatin having a rather minor effect on cancer reduction in the MAP treatment. Hence, the resistance to doxorubicin matters more than the resistance to cisplatin. Since the cisplatin-resistance does not have a large impact on the effectiveness of MAP treatment, cancer cells that are resistant to both doxorubicin and cisplatin have similar dynamics to doxorubicin-resistant cancer cells (Figure 5A,C).

### 3.4. Varying Treatment Start Time

We studied the effects of delays in the starting time of treatments on the tumors’ responses to the treatments. Since the tumor’s growth rate depends on the tumor’s size, we investigated the effects of delaying the chemotherapy treatments in small, medium, and large tumors, separately. Small tumors were chosen as follows: we first chose the tumor with the smallest cancer cell population in cluster 1, then found the tumors in cluster 2 and 3 that had cancer cell populations closest to the chosen tumor from cluster 1. Medium tumors were taken to be the mean values of all patients in each cluster. For large tumors, we took the without-treatment steady state values. We plotted the dynamics of the cancer cell population in each cluster when chemotherapy was started 1 week after diagnosis, which we assume is the earliest start time, as it takes a few days to obtain biopsy results; and the dynamics of beginning 1 month, 3 months, and 6 months after the initial diagnosis.

**Figure 6:**
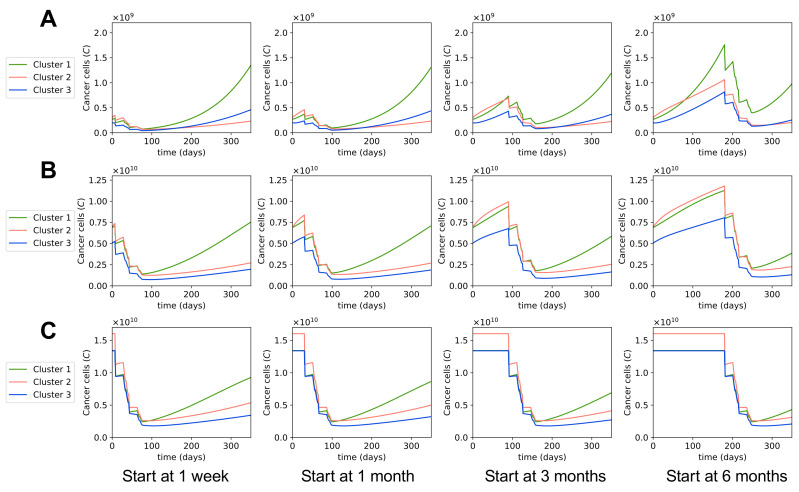
**Dynamics with different start times of MAP treatment.** Sub-figures (**A**–**C**) show the dynamics of cancer cell populations for different MAP treatment start times in small, medium, and large tumors, respectively. In each sub-figure, from left to right: the treatment started 1 week, 1 month, 3 months, or 6 months after initial diagnosis.

Figure 6 and Table 4 show that in small and medium tumors, the cancer cell populations after treatment are higher the longer we wait to start the chemotherapy. Thus, starting chemotherapy earlier leads to better outcomes with these tumors. On the contrary, the cancer cell population stays the same after treatment in large tumors regardless of the treatment start time. This is because these large tumors are at steady state and do not grow more while the patient waits for the treatment. Theoretically, the treatment start time does not matter as much for tumors at steady state or close to reaching steady state. However, in reality, when tumors are large, the functionality of the cancerous body part is likely compromised, and the quality of the patient’s life is affected, which makes us want to start the chemotherapy promptly for large tumors.

It was previously observed in [58] that tumors in cluster 1 grow fast even when the tumor is small, whereas tumors in clusters 2 and 3 start growing fast when the tumor is a bit bigger. Hence, when we delay the treatment for a long time, the small tumor in cluster 1 grows quickly and ends up with a far higher cancer cell population after the treatment than in other clusters, as seen in the treatments starting at 3 months and 6 months (Figure 6A, Table 4). For small tumors in clusters 2 and 3, even though they do grow while the patients wait for treatment, their growth rates are not as high, and their cancer cell populations after the treatment are still relatively small despite the treatment delays. Therefore, it is important to start the treatment early for small tumors of cluster 1, and it would be ideal but not as urgent to start the treatment early for small tumors in clusters 2 and 3.

Figure 6B indicates that medium-sized tumors in all three clusters grow comparably fast, and since their cancer cell populations after treatment will not be very small, we should start chemotherapy for them as early as possible. We also notice that for small and medium tumors in all clusters, the differences in cancer cell population after any treatment were not significant when comparing starting the treatment 1 week or 1 month after the diagnosis. However, treatments starting 3 or 6 months after resulted in much larger cancer cell populations after the treatment. Based on our model, it is thus not recommended to start the chemotherapy several months after the diagnosis, but rather to start within a month of the initial diagnosis.

### 3.5. Dynamics of the Cancer Microenvironment with Different Treatment Regimens

We investigated the effects of two other chemotherapy regimens on the osteosarcoma microenvironment. A combination of doxorubicin and cisplatin (AP) is a very common treatment of osteosarcoma tumors in older adults, as they are less likely to be able to tolerate high-dose methotrexate. This regimen consists of three preoperative 21-day cycles, where 25 mg/m${}^{2}$ of doxorubicin is given as a bolus once per day from day 1 to day 3, and 100 mg/m${}^{2}$ of cisplatin is given as a continuous infusion over 24 h on day 1 in each cycle [129,130]. High-dose methotrexate (MTX) has also been used as a single agent to treat osteosarcoma, with four courses of 8 to 12 mg/m${}^{2}$ given weekly before surgery [131]. In this study, we used the average dose, which is 10 mg/m${}^{2}$ of methotrexate injected over 4 h on day 1 every week for this regimen.

**Figure 7:**
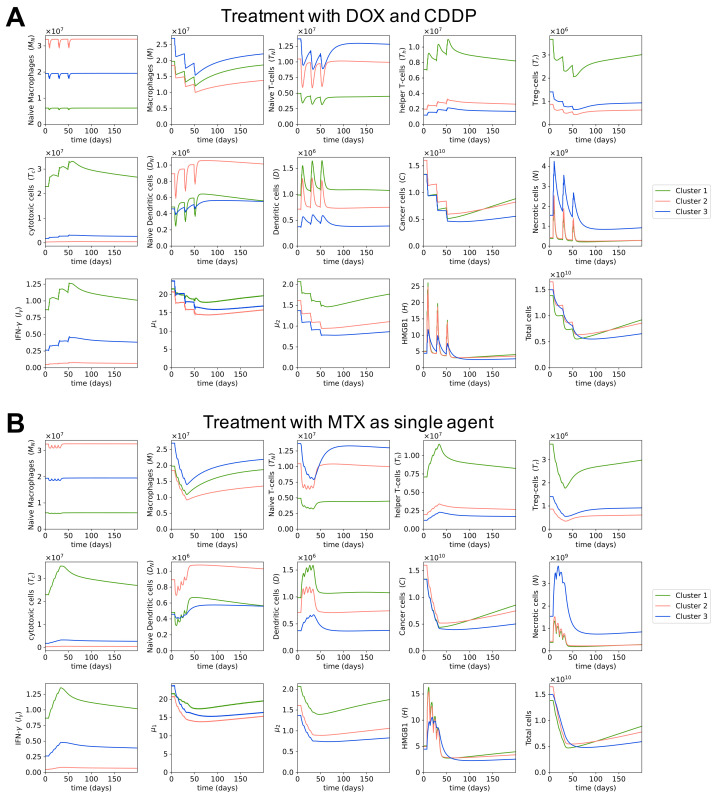
**Dynamics with different treatment regimens.** Sub-figure (**A**) shows the dynamics of cells and cytokines in osteosarcoma microenvironment in response to the combination of doxorubicin and cisplatin. Sub-figure (**B**) shows the dynamics of cells and cytokines in the osteosarcoma microenvironment in response to a high dose of methotrexate as a single agent.

Figure 7 shows that MTX and AP regimens both had higher cancer cell populations after treatment than MAP. This agrees with the finding from [132]: that the AP regimen is less effective but safer than the MAP regimen. Meanwhile, MTX as a single agent was reported to be insufficient as a neoadjuvant therapy for osteosarcoma in [131], which used the same MTX dosages and schedules as this study. Overall, according to our model, MAP is the superior treatment to MTX and AP in terms of cancer-killing ability. In fact, a recent study reports that MAP is still the favorable option for osteosarcoma among various combinations of chemotherapy drugs [133].

The AP regimen has relatively similar dynamics of cells and cytokines as the MAP regimen. That is, the populations of HMGB1, necrotic cells, and dendritic cells increase when drugs are given and decrease when no drugs are given; populations of helper T cells, cytotoxic cells, and IFN-γ decrease less than they increase, so in general they increase during treatment; and regulatory T cells, macrophages, and cytokine groups $\mu_{1}$ and $\mu_{2}$ generally decrease in abundance during treatment. The MTX treatment is given at closer intervals than AP and MAP treatments, so there is always some drug at the tumor site during MTX treatment. Therefore, the changes in cell populations and cytokine concentrations over time for MTX regimen are smoother and do not fluctuate as much as in the other two treatments, even though the dynamics of MTX regimen follow the same trend as them.

### 3.6. Optimal Dosage for MAP Treatment

Since neoadjuvant chemotherapy tries to reduce the boundaries of the tumor before surgery, we can choose the desired size of tumor for surgery and run our optimization framework to find the optimal dosages of chemotherapy drugs for the tumor to reach this size at a specific time. Osteosarcoma sizes vary greatly among patients at first diagnosis, and large tumors cannot reduce to the same size as small tumors after neoadjuvant treatment without exceeding the safe dosages of chemotherapy drugs. Thus, we chose different desired cancer cell populations to optimize for depending on the size of tumor at first diagnosis. With MAP being the preferable treatment for osteosarcoma, as mentioned in the previous section, here we present the optimal dosages of the MAP regimen for large and small tumors in each cluster; the desired cancer cell population is $2.916\times 10^{9}$ for large tumors and $1.36\times 10^{8}$ for small tumors, which is equivalent to about 5 cm per dimension (length, width, depth) for large tumors and 1.8 cm per dimension for small tumors. We used 20,000 mg/m${}^{2}$ of methotrexate, 45 mg/m${}^{2}$ of doxorubicin, and 75 mg/m${}^{2}$ of cisplatin per infusion day as maximum potential dosage, or equivalently 40,000 mg/m${}^{2}$ of methotrexate, 90 mg/m${}^{2}$ of doxorubicin, and 150 mg/m${}^{2}$ of cisplatin per 35-day cycle.

The optimal dosages for large tumors are given in Table 5. Large tumors were taken to be the steady state values of each cluster. As cluster 2 had the highest cancer cell population at the steady state, it had the highest optimal dosage for each drug of the MAP treatment among all clusters. Interestingly, clusters 1 and 3 had the same cancer cell population before treatment, but cluster 1 needed a higher dosage to achieve the same cancer cell population after the treatment as cluster 3. This was due to the fact that cluster 1’s cancer cells grew faster during treatment, so the same dosage of drugs resulted in a higher cancer cell population in cluster 1 than in cluster 3 after treatment, as seen in Section 3.1. Thus, it is important to take the tumor growth rate of the patient into account while finding the optimal dosage of chemotherapy.

**Figure 8:**
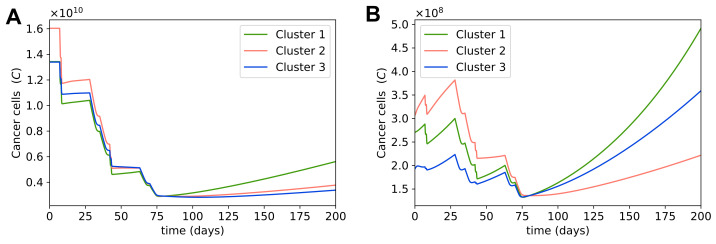
**Dynamics with optimal dosages for MAP treatment.** Sub-figure (**A**) shows the dynamics of the cancer cell population in a large tumor in each cluster; MAP dosages were optimized to obtain $2.916\times 10^{9}$ cancer cells after treatment. Sub-figure (**B**) shows the dynamics of the cancer cell population in a small tumor in each cluster; MAP dosages were optimized to obtain $1.36\times 10^{8}$ cancer cells after treatment.

Figure 8A shows that cancer cells in cluster 1 also grow fast after treatment, so it would be ideal to perform surgery quickly after neoadjuvant therapy. If it is impossible to start surgery promptly, we can choose a later time point to optimize for, so that at the time of surgery we still have the desired tumor size for resection. For example, if we cannot perform surgery until a month after chemotherapy, instead of using the cancer cell population at day 80 for optimization, which is 3 days after the second cycle of chemotherapy, we can use the cancer cell population at day 107 for optimization to find the optimal dosage, which is 30 days after the second cycle of chemotherapy. Then with the estimated optimal dosage, we will have the desired cancer cell population at day 107, which is the time of surgery.

The optimal dosages for a small tumor in each cluster are given in Table 6. Small tumors were chosen with the same method as described in Section 3.4. The cancer cell populations in these small tumors were not much larger than the desired cancer cell population after treatment, so in all clusters the optimal dosages for small tumors are much smaller than the optimal dosages for large tumors. Cluster 3 particularly, whose cancer cell population before treatment was very close to the desired cancer cell population, has very small optimal dosages.

In many cases, even though the tumor is small enough for resection, neoadjuvant chemotherapy is still given to remove any potential metastases that are too small to be yet detected. Another reason for neoadjuvant chemotherapy in small tumors is to allow evaluation of the tumor response [39]. Figure 8B suggests that although chemotherapy does not reduce cancer cell populations significantly as the cancer cell populations are already small to begin with, it helps prevent the cancer cell populations from growing bigger before surgery. Therefore, chemotherapy can also be used to control the growth ofa tumor while the patient waits for surgery.

Overall, with our optimization framework, we can find an optimal chemotherapy dosage to obtain the desired cancer cell population on the day of surgery. Our results show that it is important to consider each individual patient’s cancer growth rate while computing the optimal dosage, as patients with faster growth will need higher doses.

## 4. Discussion

Cancer is a complex disease that consists of various components, such as immune cells, lymphatic vessels, and tumor cells [134]. Traditional in vivo and in vitro studies often explore one component of cancer at a time; thus, each study alone does not supply sufficient knowledge to understand cancer in all its heterogeneity, even though these studies do provide important insights about the individual cancer components they examine [135]. Mathematical modeling has also been utilized to study the components of cancer, especially the interactions between immune cells and cancer cells. However, the majority of the models only included one or two immune cells [136,137,138,139,140,141,142,143]. Only a small subset of them have investigated the effects of multiple immune cells on cancer growth [52,53,54]. In addition, none of these studies applied their models to osteosarcoma. To the best of our knowledge, our previous work [58] was the first to study the interactions between cancer cells and the immune system in osteosarcoma.

With the increasing availability of gene expression data for many cancer types and the growing accuracy of tumor deconvolution methods, utilizing a deconvolution method on the gene expression data of a tumor to study the various components of the tumor microenvironment has become a more and more attractive option. Many recent studies applied the currently best performing deconvolution method, CIBERSORTx, to study the dynamics of cancer growth or to explore the relationships between clinical information and immune infiltrates [33,144,145,146]. In this study, we extended our previous work [58], which used CIBERSORTx to obtain immune abundances of osteosarcoma tumors and studied the tumor growth while considering its interactions with immune cells, to investigate the effects of chemotherapy on the osteosarcoma microenvironment.

Our results indicate that besides reducing the number of cancer cells, chemotherapy induces specific behaviors in certain immune cells and cytokines by causing necrosis of cancer cells. In particular, the populations of HMGB1 and dendritic cells increase when chemotherapy drugs are administered and decrease when these drugs are not given. In addition, helper T cells, cytotoxic cells, and IFN-γ generally increase in quantity during treatment, which aligns with the findings from [35,36,87,90,114,115,116,117,118,119,120,121,122,123,124,126]. Meanwhile, cells and cytokines that are not affected by this necrotic cell death decrease in abundance due to being killed by chemotherapy drugs.

We note that it is good to start chemotherapy early, unless the tumor is close to its steady state, as tumors small or medium in size will grow more while the patient waits for treatment. It is especially important to start chemotherapy promptly for tumors that grow fast, such as those in cluster 1. Interestingly, we also noticed that even with the same initial cancer cell populations and the same dosages, the cancer cell population after treatment was higher in cluster 1 than in cluster 3 (cluster 3 has slower cancer growth rate than cluster 1). All of these observations suggest that it is necessary to take the unique growth rate of the tumor into consideration when choosing the dosage and treatment start time for the patient, thereby emphasizing the importance of personalized medicine.

In this study, we introduced a simple optimization framework to find the appropriate drug dosages to achieve a desired cancer cell population on a chosen day, such as the day of surgery. The results of our optimization also agree with the above observation that the individual’s cancer growth rate is essential for calculating optimal chemotherapy dosages. Since high doses of chemotherapy are known to have high toxicity and to induce many serious health problems [147,148], it could be useful to use a mathematical model such as ours to estimate the appropriate dose rather than to give the standard dose for all tumor sizes, especially when small tumors are likely to need far smaller doses than the standard ones. Moreover, our model divides patients into groups based on their immune compositions, and thus can estimate their cancer growth more accurately than having one model for all patients, resulting in a more customized dosage recommendation for each patient.

Finding the right parameter values is a big challenge in the mathematical modeling of cancers. While it would be ideal to acquire parameters by performing in vivo and in vitro experiments, these experiments are often expensive and time-consuming. Therefore, numerous mathematical models approximate biologically reasonable values for parameters, or make assumptions about the relationships between parameters to derive their values, or vary the parameter values within certain ranges to study their effects on the outcomes. Here, we used chemotherapy-related parameters from a study that fitted these values to experimental data [84], so our treatment parameters should be more accurate than those chosen based on biological rationality or derived from assumptions. For the two parameters that we had to assume appropriate values for, we studied their impacts on the results through sensitivity analysis and by varying them, and found that different values of these parameters result in fairly similar outputs with our model.

There are still some factors that our model does not account for. For instance, there are multiple levels of cancer cell sensitivity to chemotherapy, which means two different patients can both be resistant to a chemotherapy drug, but one patient might be more sensitive than the other. Thus, the drug efficacy coefficient of doxorubicin/cisplatin-resistant cells used in our model does not represent all doxorubicin/cisplatin-resistant drug efficacies, as these parameters vary based on the levels of resistance of the cells. Despite that, our model is still useful for dose recommendations or for physicians to take into consideration while choosing between treatment options. Based on our model, a physician can monitor the tumor reduction throughout the treatment and adjust parameters such as drug efficacy coefficients according to how the tumor responds to treatment. However, it is important to note that although the results of our model align with some experimental observations in the literature, in vivo experiments should be performed to further validate our model before it can be utilized in clinic.

The chemotherapy-induced death rates of immune cells in our model were all approximated to be 0.6, so technically we could use one parameter to represent all of them. However, since chemotherapy targets cells with faster metabolic rates more successfully [89], it is reasonable to expect that the death rates via chemotherapy differ between types of immune cells. Therefore, by keeping these death rates as separate parameters, our model can be easily improved by updating the rates of chemotherapy-induced death for immune cells in proportion to their metabolic rates. Other ways to improve upon this work include adding other chemotherapy drugs, such as ifosfamide, which is also a commonly used drug for osteosarcoma [39,40]; using partial differential equations to take into account the spatial distribution of the tumors as well; or extending it to different treatment options, such as radiotherapy and immunotherapy. To use our model for another type of treatment, one has to replace chemotherapy with the treatment of interest and include the interactions between that treatment and the tumor microenvironment.

## 5. Conclusions

In this study, we developed a mathematical model for the interaction network between the most common chemotherapy drugs and the key components of osteosarcoma microenvironment. We found that during chemotherapy, dendritic cells and HMGB1 increase in population when drugs are given and decrease in population while the patient waits for the next dose of drugs. Helper T cells, cytotoxic cells, and IFN-γ increase in abundance overall. Other cells and cytokines of the microenvironment that do not succumb to necrotic cell death have reduced populations after the treatment. Overall, the MAP regimen is effective at minimizing the number of cancer cells, and works better than methotrexate alone or a combination of doxorubicin and cisplatin. Our study also suggests the importance of considering the unique growth rate of the tumor when deciding on the dosage and the treatment start time for a patient, because fast growing tumors require higher dosages and earlier starts to treatment than slow growing tumors, as shown in our results.

## Figures and Tables

**Table 1:** **Model Variables.** Names and descriptions of the variables used in the model.

Variable	Name	Description
$T_{N}$	Naive T-cells	
$T_{h}$	Helper T-cells	
$T_{C}$	Cytotoxic cells	includes CD8+ T-cells and NK cells
$T_{r}$	Regulatory T-cells	
$D_{n}$	Naive dendritic cells	
*D*	Activated dendritic cells	antigen presenting cells
$M_{N}$	Naive macrophages	includes naive macrophages and monocytes
*M*	Macrophages	includes M1 macrophages and M2 macrophages
*C*	Cancer cells	
*N*	Nectrotic cells	
*H*	HMGB1	
$\mu_{1}$	Cytokines group $\mu_{1}$	includes effects of TGF-β, IL-4, IL-10 and IL-13
$\mu_{2}$	Cytokines group $\mu_{2}$	includes effects of IL-6 and IL-17
$I_{\gamma }$	IFN-γ	
$A_{1}$	methotrexate	methotrexate concentration at tumor site
$A_{2}$	doxorubicin	doxorubicin concentration at tumor site
$A_{3}$	cisplatin	cisplatin concentration at tumor site

**Table 2:** **Chemotherapy Parameters.** Names, units, descriptions, values, and sources of chemotherapy-related parameters used in the model.

Parameter	Unit	Description	Value	Source
*f*	none	Initial fraction of cells in vulnerable phase of the cell cycle	0.5	[84]
*a*	day	Cell cycle time	0.6667	[84]
*T*	day	Duration of drug exposure		
τ	day		min(T,fa)	[84]
$\beta_{1}$	mg/L${}^{-1}$	methotrexate efficacy coefficient	2.4780	[84]
$\beta_{2}$	mg/L${}^{-1}$	doxorubicin efficacy coefficient	1.8328	[84]
$\beta_{3}$	mg/L${}^{-1}$	cisplatin efficacy coefficient	0.1467	[84]
$K_{C}$	day${}^{-1}$	Rate of chemo-induced tumor death	0.9	[89,102]
$K_{M_{N}}$	day${}^{-1}$	Rate of chemo-induced death of naive macrophages	0.6	[102]
$K_{M}$	day${}^{-1}$	Rate of chemo-induced death of macrophages	0.6	[102]
$K_{T_{N}}$	day${}^{-1}$	Rate of chemo-induced death of naive T-cells	0.6	[102]
$K_{T_{h}}$	day${}^{-1}$	Rate of chemo-induced death of helper T-cells	0.6	[102]
$K_{T_{r}}$	day${}^{-1}$	Rate of chemo-induced death of regulatory T-cells	0.6	[102]
$K_{T_{c}}$	day${}^{-1}$	Rate of chemo-induced death of cytotoxic cells	0.6	[102]
$K_{D_{N}}$	day${}^{-1}$	Rate of chemo-induced death of naive dendritic cells	0.6	[102]
$K_{D}$	day${}^{-1}$	Rate of chemo-induced death of dendritic cells	0.6	[102]
$\delta_{CT_{c}A_{3}}$	none	Effect of cisplatin to promote cancer killing ability of cytotoxic cells	1	Assumed
$\alpha_{NCA}$	none	Fraction of chemo-induced dying tumor cells that become necrotic cells	0.8	Assumed
$\delta_{A_{1}}$	day${}^{-1}$	Decay rate of methotrexate	1.4466	[106]
$\delta_{A_{2}}$	day${}^{-1}$	Decay rate of doxorubicin	8.3178	[104]
$\delta_{A_{3}}$	day${}^{-1}$	Decay rate of cisplatin	39.9253	[105]

**Table 3:** Cancer cell populations after MAP treatment with chemotherapy-resistant cells.

Cluster	Initial Cancer Population	Cancer Cell Population after Treatment			
		Chemotherapy Sensitive	Resistant to DOX	Resistant to CDDP	Resistant to DOX + CDDP
1	$1.34\times 10^{10}$	$2.44\times 10^{9}$	$3.82\times 10^{9}$	$2.49\times 10^{9}$	$3.89\times 10^{9}$
2	$1.6\times 10^{10}$	$2.6\times 10^{9}$	$4.32\times 10^{9}$	$2.66\times 10^{9}$	$4.41\times 10^{9}$
3	$1.34\times 10^{10}$	$1.87\times 10^{9}$	$3.23\times 10^{9}$	$1.92\times 10^{9}$	$3.29\times 10^{9}$

**Table 4:** Cancer cell populations after MAP treatment with different treatment start times.

Tumor Size	Cluster	Initial Cancer Population	Cancer Cell Population after Treatment			
			Start at 1 Week	Start at 1 Month	Start at 3 Months	Start at 6 Months
	1	$2.7\times 10^{8}$	$7.64\times 10^{7}$	$9.81\times 10^{7}$	$1.82\times 10^{8}$	$4.04\times 10^{8}$
Small	2	$3.07\times 10^{8}$	$6.73\times 10^{7}$	$7.76\times 10^{7}$	$1.05\times 10^{8}$	$1.52\times 10^{8}$
	3	$1.93\times 10^{8}$	$4.29\times 10^{7}$	$5.26\times 10^{7}$	$8.13\times 10^{7}$	$1.31\times 10^{8}$
	1	$6.9\times 10^{9}$	$1.41\times 10^{9}$	$1.53\times 10^{9}$	$1.81\times 10^{9}$	$2.12\times 10^{9}$
Medium	2	$6.96\times 10^{9}$	$1.27\times 10^{9}$	$1.37\times 10^{9}$	$1.6\times 10^{9}$	$1.9\times 10^{9}$
	3	$5.05\times 10^{9}$	$7.55\times 10^{8}$	$8.02\times 10^{8}$	$9.19\times 10^{8}$	$1.09\times 10^{9}$
	1	$1.34\times 10^{10}$	$2.44\times 10^{9}$	$2.44\times 10^{9}$	$2.44\times 10^{9}$	$2.44\times 10^{9}$
Large	2	$1.6\times 10^{10}$	$2.6\times 10^{9}$	$2.6\times 10^{9}$	$2.6\times 10^{9}$	$2.6\times 10^{9}$
	3	$1.34\times 10^{10}$	$1.87\times 10^{9}$	$1.87\times 10^{9}$	$1.87\times 10^{9}$	$1.87\times 10^{9}$

**Table 5:** Optimal MAP dosages for large tumors.

Cluster	Initial Cancer Population	Cancer Population after Treatment	MTX (mg/m${}^{2}$)	DOX (mg/m${}^{2}$)	CDDP (mg/m${}^{2}$)
1	$1.34\times 10^{10}$	$2.916\times 10^{9}$	8993	28	45
2	$1.6\times 10^{10}$	$2.916\times 10^{9}$	10,134	32	51
3	$1.34\times 10^{10}$	$2.916\times 10^{9}$	6176	19	31

**Table 6:** Optimal MAP dosages for small tumors.

Cluster	Initial Cancer Cell Population	Cancer Cell Population after Treatment	MTX (mg/m${}^{2}$)	DOX (mg/m${}^{2}$)	CDDP (mg/m${}^{2}$)
1	$2.7\times 10^{8}$	$1.36\times 10^{8}$	4926	15	25
2	$3.07\times 10^{8}$	$1.36\times 10^{8}$	4196	13	21
3	$1.93\times 10^{8}$	$1.36\times 10^{8}$	1305	3	6

## Data Availability

Publicly available TARGET data were downloaded from UCSC Xena web portal: https://xenabrowser.net/datapages/ (accessed on 7 July 2021). Python scripts for computations and plotting the dynamical results are available here: https://github.com/ShahriyariLab/Investigating-optimal-chemotherapy-options-for-osteosarcoma-patients-through-a-data-driven-mathemati (accessed on 7 July 2021).

## Abbreviations

The following abbreviations are used in this manuscript:

MAP	Methotrexate, doxorubicin, and cisplatin combination treatment
AP	Doxorubicin and cisplatin combination treatment
MTX	Methotrexate
DOX	Doxorubicin
CDDP	Cisplatin
TARGET	Therapeutically Applicable Research to Generate Effective Treatments
ODE	Ordinary differential equation
HMGB1	High mobility group box 1
DAMP	Damage-associated molecular pattern
NK	Natural killer

## Appendix A. ODE System and Non-Dimensionalization

(A1) $$\begin{array}{cc} \frac{d\left[M_{N}\right]}{dt}=\; & A_{M_{N}}-\left(\lambda_{MI_{\gamma }}\left[I_{\gamma }\right]+\lambda_{M\mu_{1}}\left[\mu_{1}\right]\right)\left[M_{N}\right]-\delta_{M_{N}}\left[M_{N}\right]\\ \; & -(K_{M_{N}}\left(f-\frac{\tau }{a}+\frac{1}{24a}\right)\left(1-e^{-\beta_{1}A_{1}}\right)+K_{M_{N}}\left(1-e^{-\beta_{2}A_{2}}\right)\\ \; & +K_{M_{N}}\left(1-e^{-\beta_{3}A_{3}}\right))\left[M_{N}\right] \end{array}$$

(A2) $$\begin{array}{cc} \frac{d\left[M\right]}{dt}=\; & \left(\lambda_{MI_{\gamma }}\left[I_{\gamma }\right]+\lambda_{M\mu_{1}}\left[\mu_{1}\right]\right)\left[M_{N}\right]-\delta_{M}\left[M\right]-(K_{M}\left(f-\frac{\tau }{a}+\frac{1}{24a}\right)\left(1-e^{-\beta_{1}A_{1}}\right)\\ \; & +K_{M}\left(1-e^{-\beta_{2}A_{2}}\right)+K_{M}\left(1-e^{-\beta_{3}A_{3}}\right))\left[M\right] \end{array}$$

(A3) $$\begin{array}{cc} \frac{d\left[T_{N}\right]}{dt}=\; & A_{T_{N}}-\left(\lambda_{T_{h}M}\left[M\right]+\lambda_{T_{h}D}\left[D\right]\right)\left[T_{N}\right]-\lambda_{T_{r}\mu_{1}}\left[\mu_{1}\right]\left[T_{N}\right]\\ \; & -\left(\lambda_{T_{c}T_{h}}\left[T_{h}\right]+\lambda_{T_{c}M}\left[M\right]+\lambda_{T_{c}D}\left[D\right]\right)\left[T_{N}\right]-\delta_{T_{N}}\left[T_{N}\right]\\ \; & -(K_{T_{N}}\left(f-\frac{\tau }{a}+\frac{1}{24a}\right)\left(1-e^{-\beta_{1}A_{1}}\right)+K_{T_{N}}\left(1-e^{-\beta_{2}A_{2}}\right)\\ \; & +K_{T_{N}}\left(1-e^{-\beta_{3}A_{3}}\right))\left[T_{N}\right] \end{array}$$

(A4) $$\begin{array}{cc} \frac{d\left[T_{h}\right]}{dt}=\; & \left(\lambda_{T_{h}M}\left[M\right]+\lambda_{T_{h}D}\left[D\right]\right)\left[T_{N}\right]-\left(\delta_{T_{h}T_{r}}\left[T_{r}\right]+\delta_{T_{h}\mu_{1}}\left[\mu_{1}\right]+\delta_{T_{h}}\right)\left[T_{h}\right]\\ \; & -(K_{T_{h}}\left(f-\frac{\tau }{a}+\frac{1}{24a}\right)\left(1-e^{-\beta_{1}A_{1}}\right)+K_{T_{h}}\left(1-e^{-\beta_{2}A_{2}}\right)\\ \; & +K_{T_{h}}\left(1-e^{-\beta_{3}A_{3}}\right))\left[T_{h}\right] \end{array}$$

(A5) $$\begin{array}{cc} \frac{d\left[T_{r}\right]}{dt}=\; & \left(\lambda_{T_{r}\mu_{1}}\left[\mu_{1}\right]\right)\left[T_{N}\right]-\delta_{T_{r}}\left[T_{r}\right]-(K_{T_{r}}\left(f-\frac{\tau }{a}+\frac{1}{24a}\right)\left(1-e^{-\beta_{1}A_{1}}\right)\\ \; & +K_{T_{r}}\left(1-e^{-\beta_{2}A_{2}}\right)+K_{T_{r}}\left(1-e^{-\beta_{3}A_{3}}\right))\left[T_{r}\right] \end{array}$$

(A6) $$\begin{array}{cc} \frac{d\left[T_{c}\right]}{dt}=\; & \left(\lambda_{T_{c}T_{h}}\left[T_{h}\right]+\lambda_{T_{c}M}\left[M\right]+\lambda_{T_{c}D}\left[D\right]\right)\left[T_{N}\right]-\left(\delta_{T_{c}T_{r}}\left[T_{r}\right]+\delta_{T_{c}\mu_{1}}\left[\mu_{1}\right]+\delta_{T_{c}}\right)\left[T_{c}\right]\\ \; & -(K_{T_{c}}\left(f-\frac{\tau }{a}+\frac{1}{24a}\right)\left(1-e^{-\beta_{1}A_{1}}\right)+K_{T_{c}}\left(1-e^{-\beta_{2}A_{2}}\right)\\ \; & +K_{T_{c}}\left(1-e^{-\beta_{3}A_{3}}\right))\left[T_{c}\right] \end{array}$$

(A7) $$\begin{array}{cc} \frac{d\left[D_{N}\right]}{dt}=\; & A_{D_{N}}-\left(\lambda_{DC}\left[C\right]+\lambda_{DH}\left[H\right]\right)\left[D_{N}\right]-\delta_{D_{N}}\left[D_{N}\right]\\ \; & -(K_{D_{N}}\left(f-\frac{\tau }{a}+\frac{1}{24a}\right)\left(1-e^{-\beta_{1}A_{1}}\right)+K_{D_{N}}\left(1-e^{-\beta_{2}A_{2}}\right)\\ \; & +K_{D_{N}}\left(1-e^{-\beta_{3}A_{3}}\right))\left[D_{N}\right] \end{array}$$

(A8) $$\begin{array}{cc} \frac{d\left[D\right]}{dt}=\; & \left(\lambda_{DC}\left[C\right]+\lambda_{DH}\left[H\right]\right)\left[D_{N}\right]-\left(\delta_{DC}\left[C\right]+\delta_{D}\right)\left[D\right]\\ \; & -(K_{D}\left(f-\frac{\tau }{a}+\frac{1}{24a}\right)\left(1-e^{-\beta_{1}A_{1}}\right)+K_{D}\left(1-e^{-\beta_{2}A_{2}}\right)\\ \; & +K_{D}\left(1-e^{-\beta_{3}A_{3}}\right))\left[D\right] \end{array}$$

(A9) $$\begin{array}{cc} \frac{d\left[C\right]}{dt}=\; & \left(\lambda_{C}+\lambda_{C\mu_{1}}\left[\mu_{1}\right]+\lambda_{C\mu_{2}}\left[\mu_{2}\right]\right)\left[C\right]\left(1-\frac{\left[C\right]}{C_{0}}\right)\\ \; & -(\delta_{CI_{\gamma }}\left[I_{\gamma }\right]+\delta_{C}+\delta_{CT_{c}}\left(1+\delta_{CT_{c}A_{3}}\left(1-e^{-\beta_{3}A_{3}}\right)\right)\left[T_{c}\right])\left[C\right]\\ \; & -(K_{C}\left(f-\frac{\tau }{a}+\frac{1}{24a}\right)\left(1-e^{-\beta_{1}A_{1}}\right)+K_{C}\left(1-e^{-\beta_{2}A_{2}}\right)\\ \; & +K_{C}\left(1-e^{-\beta_{3}A_{3}}\right))\left[C\right] \end{array}$$

(A10) $$\begin{array}{cc} \frac{d\left[N\right]}{dt}=\; & \alpha_{NC}(\delta_{CI_{\gamma }}\left[I_{\gamma }\right]+\delta_{C}+\delta_{CT_{c}}\left(1+\delta_{CT_{c}A_{3}}\left(1-e^{-\beta_{3}A_{3}}\right)\right)\left[T_{c}\right])\left[C\right]\\ \; & +\alpha_{\mathrm{NCA}}(K_{C}\left(f-\frac{\tau }{a}+\frac{1}{24a}\right)\left(1-e^{-\beta_{1}A_{1}}\right)+K_{C}\left(1-e^{-\beta_{2}A_{2}}\right)\\ \; & +K_{C}\left(1-e^{-\beta_{3}A_{3}}\right))\left[C\right]-\delta_{N}\left[N\right] \end{array}$$

(A11) $$\begin{array}{cc} \frac{d\left[I_{\gamma }\right]}{dt}=\; & \lambda_{I_{\gamma }T_{h}}\left[T_{h}\right]+\lambda_{I_{\gamma }T_{c}}\left[T_{c}\right]-\delta_{I_{\gamma }}\left[I_{\gamma }\right] \end{array}$$

(A12) $$\begin{array}{cc} \frac{d\left[\mu_{1}\right]}{dt}=\; & \lambda_{\mu_{1}T_{h}}\left[T_{h}\right]+\lambda_{\mu_{1}M}\left[M\right]+\lambda_{\mu_{1}C}\left[C\right]-\delta_{\mu_{1}}\left[\mu_{1}\right] \end{array}$$

(A13) $$\begin{array}{cc} \frac{d\left[\mu_{2}\right]}{dt}=\; & \lambda_{\mu_{2}T_{h}}\left[T_{h}\right]+\lambda_{\mu_{2}M}\left[M\right]+\lambda_{\mu_{2}C}\left[C\right]-\delta_{\mu_{2}}\left[\mu_{2}\right] \end{array}$$

(A14) $$\begin{array}{cc} \frac{d\left[H\right]}{dt}=\; & \lambda_{HM}\left[M\right]+\lambda_{HD}\left[D\right]+\lambda_{HN}\left[N\right]-\delta_{H}\left[H\right] \end{array}$$

(A15) $$\begin{array}{cc} \frac{d[A_{1}]}{\mathrm{dt}}=\; & v_{A_{1}}\left(t\right)-\delta_{A_{1}}\left[A_{1}\right] \end{array}$$

(A16) $$\begin{array}{cc} \frac{d[A_{2}]}{\mathrm{dt}}=\; & v_{A_{2}}\left(t\right)-\delta_{A_{2}}\left[A_{2}\right] \end{array}$$

(A17) $$\begin{array}{cc} \frac{d[A_{3}]}{\mathrm{dt}}=\; & v_{A_{3}}\left(t\right)-\delta_{A_{3}}\left[A_{3}\right] \end{array}$$

(A18) $$\begin{array}{cc} \frac{d\left[{\bar{M}}_{N}\right]}{dt}=\; & {\bar{A}}_{M_{N}}-\alpha_{M_{N}M}\left({\bar{\lambda }}_{MI_{\gamma }}\left[{\bar{I}}_{\gamma }\right]+{\bar{\lambda }}_{M\mu_{1}}\left[{\bar{\mu }}_{1}\right]\right)\left[{\bar{M}}_{N}\right]-\delta_{M_{N}}\left[{\bar{M}}_{N}\right]\\ \; & -(K_{M_{N}}\left(f-\frac{\tau }{a}+\frac{1}{24a}\right)\left(1-e^{-{\bar{\beta }}_{1}{\bar{A}}_{1}}\right)+K_{M_{N}}\left(1-e^{-{\bar{\beta }}_{2}{\bar{A}}_{2}}\right)\\ \; & +K_{M_{N}}\left(1-e^{-{\bar{\beta }}_{3}{\bar{A}}_{3}}\right))\left[{\bar{M}}_{N}\right] \end{array}$$

We introduce the following new non-dimensional variables:

$$\begin{array}{cccccc} {\bar{A}}_{1}=\; & \frac{A_{1}\delta_{A_{1}}}{v_{A_{1}}^{*}},\; & {\bar{A}}_{2}=\; & \frac{A_{2}\delta_{A_{2}}}{v_{A_{2}}^{*}},\; & {\bar{A}}_{3}=\; & \frac{A_{3}\delta_{A_{3}}}{v_{A_{3}}^{*}} \end{array}$$

Non-dimensional system (only equations with changes):

(A19) $$\begin{array}{cc} \frac{d\left[\bar{M}\right]}{dt}=\; & \left({\bar{\lambda }}_{MI_{\gamma }}\left[{\bar{I}}_{\gamma }\right]+{\bar{\lambda }}_{M\mu_{1}}\left[{\bar{\mu }}_{1}\right]\right)\left[{\bar{M}}_{N}\right]-\delta_{M}\left[\bar{M}\right]-(K_{M}\left(f-\frac{\tau }{a}+\frac{1}{24a}\right)\left(1-e^{-{\bar{\beta }}_{1}{\bar{A}}_{1}}\right)\\ \; & +K_{M}\left(1-e^{-{\bar{\beta }}_{2}{\bar{A}}_{2}}\right)+K_{M}\left(1-e^{-{\bar{\beta }}_{3}{\bar{A}}_{3}}\right))\left[\bar{M}\right] \end{array}$$

(A20) $$\begin{array}{cc} \frac{d\left[{\bar{T}}_{N}\right]}{dt}=\; & {\bar{A}}_{T_{N}}-\alpha_{T_{N}T_{h}}\left({\bar{\lambda }}_{T_{h}M}\left[\bar{M}\right]+{\bar{\lambda }}_{T_{h}D}\left[\bar{D}\right]\right)\left[{\bar{T}}_{N}\right]-\alpha_{T_{N}T_{r}}{\bar{\lambda }}_{T_{r}\mu_{1}}\left[{\bar{\mu }}_{1}\right]\left[{\bar{T}}_{N}\right]\\ \; & -\alpha_{T_{N}T_{c}}\left({\bar{\lambda }}_{T_{c}T_{h}}\left[{\bar{T}}_{h}\right]+{\bar{\lambda }}_{T_{c}M}\left[\bar{M}\right]+{\bar{\lambda }}_{T_{c}D}\left[\bar{D}\right]\right)\left[{\bar{T}}_{N}\right]-\delta_{T_{N}}\left[{\bar{T}}_{N}\right]\\ \; & -(K_{T_{N}}\left(f-\frac{\tau }{a}+\frac{1}{24a}\right)\left(1-e^{-{\bar{\beta }}_{1}{\bar{A}}_{1}}\right)+K_{T_{N}}\left(1-e^{-{\bar{\beta }}_{2}{\bar{A}}_{2}}\right)\\ \; & +K_{T_{N}}\left(1-e^{-{\bar{\beta }}_{3}{\bar{A}}_{3}}\right))\left[{\bar{T}}_{N}\right] \end{array}$$

(A21) $$\begin{array}{cc} \frac{d\left[{\bar{T}}_{h}\right]}{dt}=\; & \left({\bar{\lambda }}_{T_{h}M}\left[\bar{M}\right]+{\bar{\lambda }}_{T_{h}D}\left[\bar{D}\right]\right)\left[{\bar{T}}_{N}\right]-\left({\bar{\delta }}_{T_{h}T_{r}}\left[{\bar{T}}_{r}\right]+{\bar{\delta }}_{T_{h}\mu_{1}}\left[{\bar{\mu }}_{1}\right]+\delta_{T_{h}}\right)\left[{\bar{T}}_{h}\right]\\ \; & -(K_{T_{h}}\left(f-\frac{\tau }{a}+\frac{1}{24a}\right)\left(1-e^{-{\bar{\beta }}_{1}{\bar{A}}_{1}}\right)+K_{T_{h}}\left(1-e^{-{\bar{\beta }}_{2}{\bar{A}}_{2}}\right)\\ \; & +K_{T_{h}}\left(1-e^{-{\bar{\beta }}_{3}{\bar{A}}_{3}}\right))\left[{\bar{T}}_{h}\right] \end{array}$$

(A22) $$\begin{array}{cc} \frac{d\left[{\bar{T}}_{r}\right]}{dt}=\; & \left({\bar{\lambda }}_{T_{r}\mu_{1}}\left[{\bar{\mu }}_{1}\right]\right)\left[{\bar{T}}_{N}\right]-\delta_{T_{r}}\left[{\bar{T}}_{r}\right]-(K_{T_{r}}\left(f-\frac{\tau }{a}+\frac{1}{24a}\right)\left(1-e^{-{\bar{\beta }}_{1}{\bar{A}}_{1}}\right)\\ \; & +K_{T_{r}}\left(1-e^{-{\bar{\beta }}_{2}{\bar{A}}_{2}}\right)+K_{T_{r}}\left(1-e^{-{\bar{\beta }}_{3}{\bar{A}}_{3}}\right))\left[{\bar{T}}_{r}\right] \end{array}$$

(A23) $$\begin{array}{cc} \frac{d\left[{\bar{T}}_{c}\right]}{dt}=\; & \left({\bar{\lambda }}_{T_{c}T_{h}}\left[{\bar{T}}_{h}\right]+{\bar{\lambda }}_{T_{c}M}\left[\bar{M}\right]+{\bar{\lambda }}_{T_{c}D}\left[\bar{D}\right]\right)\left[{\bar{T}}_{N}\right]-\left({\bar{\delta }}_{T_{c}T_{r}}\left[{\bar{T}}_{r}\right]+{\bar{\delta }}_{T_{c}\mu_{1}}\left[{\bar{\mu }}_{1}\right]+\delta_{T_{c}}\right)\left[{\bar{T}}_{c}\right]\\ \; & -(K_{T_{c}}\left(f-\frac{\tau }{a}+\frac{1}{24a}\right)\left(1-e^{-{\bar{\beta }}_{1}{\bar{A}}_{1}}\right)+K_{T_{c}}\left(1-e^{-{\bar{\beta }}_{2}{\bar{A}}_{2}}\right) \end{array}$$

(A24) $$\begin{array}{cc} \frac{d\left[{\bar{D}}_{N}\right]}{dt}=\; & {\bar{A}}_{D_{N}}-\alpha_{D_{N}D}\left({\bar{\lambda }}_{DC}\left[\bar{C}\right]+{\bar{\lambda }}_{DH}\left[\bar{H}\right]\right)\left[{\bar{D}}_{N}\right]-\delta_{D_{N}}\left[{\bar{D}}_{N}\right]\\ \; & -(K_{D_{N}}\left(f-\frac{\tau }{a}+\frac{1}{24a}\right)\left(1-e^{-{\bar{\beta }}_{1}{\bar{A}}_{1}}\right)+K_{D_{N}}\left(1-e^{-{\bar{\beta }}_{2}{\bar{A}}_{2}}\right)\\ \; & +K_{D_{N}}\left(1-e^{-{\bar{\beta }}_{3}{\bar{A}}_{3}}\right))\left[{\bar{D}}_{N}\right] \end{array}$$

(A25) $$\begin{array}{cc} \frac{d\left[\bar{D}\right]}{dt}=\; & \left({\bar{\lambda }}_{DC}\left[\bar{C}\right]+{\bar{\lambda }}_{DH}\left[\bar{H}\right]\right)\left[{\bar{D}}_{N}\right]-\left({\bar{\delta }}_{DC}\left[\bar{C}\right]+\delta_{D}\right)\left[\bar{D}\right]\\ \; & -(K_{D}\left(f-\frac{\tau }{a}+\frac{1}{24a}\right)\left(1-e^{-{\bar{\beta }}_{1}{\bar{A}}_{1}}\right)+K_{D}\left(1-e^{-{\bar{\beta }}_{2}{\bar{A}}_{2}}\right)\\ \; & +K_{D}\left(1-e^{-{\bar{\beta }}_{3}{\bar{A}}_{3}}\right))\left[\bar{D}\right] \end{array}$$

(A26) $$\begin{array}{cc} \frac{d\left[\bar{C}\right]}{dt}=\; & \left(\lambda_{C}+{\bar{\lambda }}_{C\mu_{1}}\left[{\bar{\mu }}_{1}\right]+{\bar{\lambda }}_{C\mu_{2}}\left[{\bar{\mu }}_{2}\right]\right)\left[\bar{C}\right]\left(1-\frac{\left[\bar{C}\right]}{{\bar{C}}_{0}}\right)\\ \; & -\left({\bar{\delta }}_{CI_{\gamma }}\left[{\bar{I}}_{\gamma }\right]+\delta_{C}+{\bar{\delta }}_{CT_{c}}\left(1+{\bar{\delta }}_{CT_{c}A_{3}}\left(1-e^{-{\bar{\beta }}_{3}{\bar{A}}_{3}}\right)\right)\left[{\bar{T}}_{c}\right]\right)\left[\bar{C}\right]\\ \; & -(K_{C}\left(f-\frac{\tau }{a}+\frac{1}{24a}\right)\left(1-e^{-{\bar{\beta }}_{1}{\bar{A}}_{1}}\right)+K_{C}\left(1-e^{-{\bar{\beta }}_{2}{\bar{A}}_{2}}\right)\\ \; & +K_{C}\left(1-e^{-{\bar{\beta }}_{3}{\bar{A}}_{3}}\right))\left[\bar{C}\right] \end{array}$$

(A27) $$\begin{array}{cc} \frac{d\left[\bar{N}\right]}{dt}=\; & {\bar{\alpha }}_{NC}\left({\bar{\delta }}_{CI_{\gamma }}\left[{\bar{I}}_{\gamma }\right]+\delta_{C}+{\bar{\delta }}_{CT_{c}}\left(1+{\bar{\delta }}_{CT_{c}A_{3}}\left(1-e^{-{\bar{\beta }}_{3}{\bar{A}}_{3}}\right)\right)\left[{\bar{T}}_{c}\right]\right)\left[\bar{C}\right]\\ \; & +{\bar{\alpha }}_{\mathrm{NCA}}(K_{C}\left(f-\frac{\tau }{a}+\frac{1}{24a}\right)\left(1-e^{-{\bar{\beta }}_{1}{\bar{A}}_{1}}\right)+K_{C}\left(1-e^{-{\bar{\beta }}_{2}{\bar{A}}_{2}}\right)\\ \; & +K_{C}\left(1-e^{-{\bar{\beta }}_{3}{\bar{A}}_{3}}\right))\left[\bar{C}\right]-\delta_{N}\left[\bar{N}\right] \end{array}$$

(A28) $$\begin{array}{cc} \frac{d[{\bar{A}}_{1}]}{\mathrm{dt}}=\; & {\bar{v}}_{A_{1}}\left(t\right)-\delta_{A_{1}}\left[{\bar{A}}_{1}\right] \end{array}$$

(A29) $$\begin{array}{cc} \frac{d[{\bar{A}}_{2}]}{\mathrm{dt}}=\; & {\bar{v}}_{A_{2}}\left(t\right)-\delta_{A_{2}}\left[{\bar{A}}_{2}\right] \end{array}$$

(A30) $$\begin{array}{cc} \frac{d[{\bar{A}}_{3}]}{\mathrm{dt}}=\; & {\bar{v}}_{A_{3}}\left(t\right)-\delta_{A_{3}}\left[{\bar{A}}_{3}\right] \end{array}$$

where the new non-dimensional parameters are:

$$\begin{array}{cccccc} {\bar{\beta }}_{1}=\; & \frac{\beta_{1}v_{A_{1}}^{*}}{\delta_{A_{1}}},\; & {\bar{\beta }}_{2}=\; & \frac{\beta_{2}v_{A_{2}}^{*}}{\delta_{A_{2}}},\; & {\bar{\beta }}_{3}=\; & \frac{\beta_{3}v_{A_{3}}^{*}}{\delta_{A_{3}}},\\ {\bar{\delta }}_{CT_{c}A_{3}}=\; & \delta_{CT_{c}A_{3}}T_{c}^{\infty },\; & {\bar{\alpha }}_{NCA}=\; & \alpha_{NCA}\frac{C^{\infty }}{N^{\infty }},\; & {\bar{v}}_{A_{1}}\left(t\right)=\; & \frac{v_{A_{1}}\left(t\right)\delta_{A_{1}}}{v_{A_{1}}^{*}},\\ {\bar{v}}_{A_{2}}\left(t\right)=\; & \frac{v_{A_{2}}\left(t\right)\delta_{A_{2}}}{v_{A_{2}}^{*}},\; & {\bar{v}}_{A_{3}}\left(t\right)=\; & \frac{v_{A_{3}}\left(t\right)\delta_{A_{3}}}{v_{A_{3}}^{*}}, \end{array}$$
